# A New Survival Model Based on ADAMTSs for Prognostic Prediction in Clear Cell Renal Cell Carcinoma

**DOI:** 10.1155/2021/2606213

**Published:** 2021-09-23

**Authors:** Guangzhen Wu, Jianyi Li, Yingkun Xu, Xiangyu Che, Feng Chen, Qifei Wang

**Affiliations:** ^1^Department of Urology, The First Affiliated Hospital of Dalian Medical University, Dalian, Liaoning, China; ^2^Organ Transplant Center, The First Affiliated Hospital, Sun Yat-sen University, Guangzhou, China; ^3^Guangdong Provincial Key Laboratory on Organ Donation and Transplant Immunology, Guangzhou, China; ^4^Department of Endocrine and Breast Surgery, The First Affiliated Hospital of Chongqing Medical University, Chongqing, China

## Abstract

The main purpose of this study was to explore the genetic variation, gene expression, and clinical significance of ADAMTSs (a disintegrin and metalloprotease domains with thrombospondin motifs) across cancer types. Analysis of data from the TCGA (The Cancer Genome Atlas) database showed that the ADAMTSs have extensive CNV (copy number variation) and SNV (single nucleotide variation) across cancer types. Compared with normal tissues, the methylation of ADAMTSs in cancer tissues is also significantly different, which affects the expression of ADAMTS gene and the prognosis of cancer patients. Through gene expression analysis, we found that ADAMTS family has significant changes in gene expression across cancer types and is closely related to the prognosis of carcinoma, especially in ccRCC (clear cell renal cell carcinoma). LASSO regression analysis was used to establish a prognostic model based on the ADAMTSs to judge the prognosis of patients with ccRCC. Multiple Cox regression analysis suggested that age, grade, stage, and risk score of the prognostic model of ccRCC were independent prognostic factors in patients with renal clear cell carcinoma. These findings indicate that the ADAMTSs-based survival model can accurately predict the prognosis of patients with ccRCC and suggest that ADAMTSs are a potential prognostic biomarker and therapeutic target in ccRCC.

## 1. Introduction

In 1997, the first member of the ADAMTS family was found in a colon cancer patient [1]. To indicate that there are three thrombospondin type-1 (TS1) motifs in its structure, the enzyme is named ADAMTS1. The discovery of ADAMTS1 promotes the emergence of other new ADAMTS genes, and the emergence of the human genome sequence led to the completion of 19 human ADAMTS molecular clones in 2003 [2, 3]. The discovery of genes encoding ADAMTS-like proteins has made the genetic catalog of this superfamily more complete [4].

ADAMTS enzymes play an important role in tissue morphogenesis and patho-physiological remodeling, inflammation, and vascular biology [5]. Studies have shown that mutations in genes such as ADAMTSL2, ADAMTSL4, ADAMTS2, ADAMTS10, ADAMTS13, and ADAMTS17 could lead to genetic diseases, and there is a potential synergy between ADAMTS proteins [6]. This connection is undoubtedly a very important news for cancer workers. As we all know, tumorigenesis is closely related to genetics and genes. More and more studies have shown that the ADAMTS is closely related to cancer. ADAMTSs affect cell proliferation, adhesion, migration, and angiogenesis by cleavage or interaction with a variety of extracellular matrix components or regulatory factors, thus affecting tumor development and prognosis [7].

Many studies have shown that ADAMTSs play an important role in a variety of tumors, including gastrointestinal tumors [8], breast cancer [9, 10], epithelial ovarian cancer [11], and renal cell carcinoma [12, 13]. Considering the different roles of ADAMTSs in tumors, some play an antitumor role and some play a tumor protective role, so it is necessary to analyze ADAMTSs as a whole. But so far, to our knowledge, there has not been a relatively overall study of ADAMTSs across cancer types. The main purpose of this research was to study the mutation and expression of ADAMTSs in 32 kinds of tumors to establish a prognostic model of KIRC (kidney renal cell carcinoma) based on ADAMTSs and to analyze the main pathways through which ADAMTSs play a role.

## 2. Materials and Methods

### 2.1. Data Acquisition

The raw data for our study came from the TCGA database (https://cancergenome.nih.gov/). 32 different TCGA datasets were analyzed, each dataset representing a specific type of cancer. Through this database, we downloaded CNV and SNV data of 32 kinds of cancers and analyzed them with Perl language; TBtools software was used to visualize them [14]. The RNA-seq transcriptome data of the KIRC group were downloaded from the Genomic Data Commons (GDC) portal through R/Bioconductor package TCGAbiolink [15], including 72 normal renal specimens and 539 KIRC specimens. The expression data of ADAMTSs in 539 cases of KIRC tissue and 72 cases of normal renal tissue were analyzed by Limma package, and the heat map of ADAMTSs was visualized by TBtools software. Cancer patients' clinical information came from TCGAbiolink, including tumor size status (T), metastatic status (M), tumor grade, tumor stage, and age and survival status. Then, Perl language and Rstudio were used to analyze the data. Lasso regression analysis was carried out with “Glmnet” and “Survival” packages. “Survival” package was used for univariate and multivariate Cox risk analysis of clinical features.

### 2.2. The Differential Expression between Tumor and Adjacent Normal Tissues for Gene across Cancer Types

GSCALite database was used for the analysis of the expression data of ADAMTSs across cancer types (http://bioinfo.life.hust.edu.cn/web/GSCA.Lite/) [16]. The differential expression between carcinoma tissues and adjacent normal tissues for ADAMTS20 and ADAMTS14 across cancer types was analyzed through TIMER website (https://cistrome.shinyapps.io/timer/) [17]. ADAMTS20 immunohistochemical staining images of ccRCC and normal kidney tissue were obtained from the Human Protein Atlas website (https://www.proteinatlas.org/) [18].

### 2.3. PPI Networks and Coexpression of ADAMTSs

STRING online tool (https://string-db.org/cgi/) [19] was used to analyze the interactions between 24 proteins of the ADAMTSs and visualized them with Cytoscape (http://www.cytoscape.org/) [20]. The coexpression among the members of ADAMTSs was analyzed by “Corrplot” package.

### 2.4. The Analysis of Genomic Variation, Methylation Changes, Classical Pathways, and Drug Sensitivity

We used GSCALite database to analyze the difference of ADAMTS methylation between tumors and normal tissues, the relationship between methylation and expression, and the relationship between methylation and survival. The degree of activation or inhibition of classical pathway by ADAMTSs was analyzed by GSCALite database. Survival analysis of ADAMTSs and drug sensitivity analysis were also done with GSCALite database.

### 2.5. Construction of Regression Model and Risk Score

The construction of regression model and risk score refers to the construction of prognostic model of ferroptosis-related genes [21]. We used univariate Cox models to analyze the correlation between overall survival (OS) and ADAMTS expression level of patients with KIRC. We used lasso regression analysis to eliminate genes that may overfit the model. Finally, multivariate analysis was used to determine the optimal predictive factor ADAMTS of the model. The number of genes is expressed by *N*, Coei represents the coefficient value, and Expi represents the of gene expression level. We took the median as the cut-off value, according to which all patients with KIRC were divided into two groups: low-risk and high-risk groups. The overall survival time-dependent recipient operating characteristics were applied to evaluate the accuracy of the prognostic model.

### 2.6. Statistical Analyses

Statistical significance of differential expression from TIMER website was evaluated using the Wilcoxon test. We applied one-way ANOVA to compare the expression of ADAMTSs in carcinoma tissues and normal tissues. We applied student's *t*-test to compare the expression of ADAMTSs in KIRC datasets according to age, stage, grade, and *T* and *M* status. “Survminer” package was used to determine the cut-off value of each risk score of carcinoma group, and according to the best cut-off value, the patients were divided into high-risk group and low-risk group. *P* < 0.05 was considered to be statistically significant.

## 3. Results

### 3.1. Extensive Genetic Changes of ADAMTS in 32 Cancer Types

**Figure 1:**
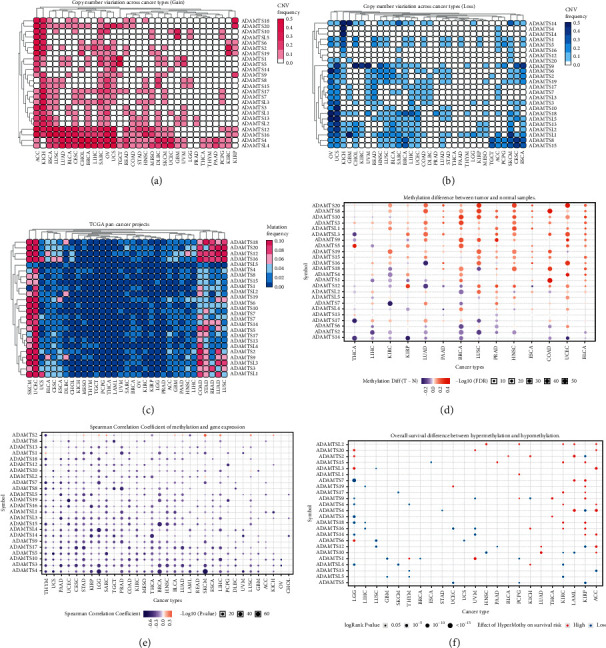
Genetic changes in ADAMTSs across cancer types. (a) The CNV frequency of the ADAMTSs (gain). (b) The CNV frequency of the ADAMTSs (loss). (c) The SNV frequency of the ADAMTSs. (d) Difference of methylation of ADAMTSs between tumor and normal tissue. (e) Correlation between methylation of ADAMTSs and gene expression. (f) The relationship between methylation of ADAMTSs and overall survival rate.

We conducted a comprehensive literature review and identified 24 key ADAMTS genes. We then used the TCGA database to determine the CNV of the 24 ADAMTSs across 32 cancer types. The raw ADAMTS CNV data of 32 tumors were downloaded from TCGA database, and then we analyzed them with Perl and R language and visualized them with TBtools. We found that there were varying degrees of gain or lost copy number variation of ADAMTS protein in 32 tumors (Figures 1(a) and 1(b), Tables S1 and S2). ADAMTS genes have higher copy number gain in ACC and KICH, and ADAMTS4, ADAMTS12, ADAMTS16, and ADAMTSL4 have higher probability of copy number gain in different tumors. ADAMTS genes have high copy number loss in OV and UCS. ADAMTSL1 and ADAMTS18 have a higher probability of copy number loss in different tumors. Through the analysis of single nucleotide variants, we found that ADAMTS genes have varying degrees of single nucleotide variants in 32 kinds of tumors, of which the mutation in SKCM and UCEC is relatively high (Figure 1(c), Table S3). At the same time, we analyzed the difference of ADAMTS gene methylation between tumors and normal tissues. The results showed that there was a significant difference in ADAMTS gene methylation between tumors and normal tissues (Figure 1(d)), and there was generally a negative correlation between methylation and gene expression (Figure 1(e)). Survival analysis has shown that hypermethylation indicates a higher risk of survival (Figure 1(f)).

### 3.2. Expression of ADAMTS Gene across Cancer Types

We used R language and TBtools to analyze the mRNA expression of ADAMTSs in different types of tumors from TCGA database. The results showed that there were significant differences in the expression of ADAMTS gene between different types of tumors and normal tissues (Figure S1A and Tables S4 and S5), especially ADAMTS20 (Figure S1B) and ADAMTS14 (Figure S1C) were significantly overexpressed in many kinds of tumors. The analysis of the Human Protein Atlas database also showed that the expression of ADAMTS20 in clear cell renal cell carcinoma was significantly increased (Figures S1D and S1E), which was consistent with the results of previous studies.

### 3.3. The Connection between ADAMTS Families

**Figure 2:**
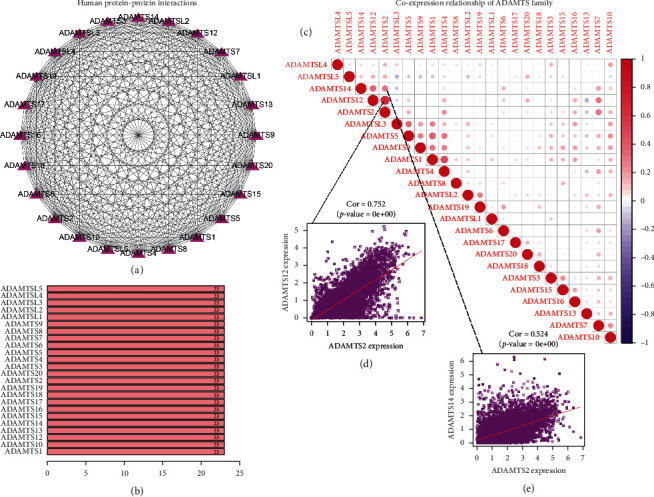
PPI network of ADAMTSs: (a) the PPI network analysis results of ADAMTSs. (b) Quantitative maps of PPI between ADAMTS genes. (c–e) Coexpression analysis showed that there was a correlation between the expressions of ADAMTSs.

In order to better explore the relationship between ADAMTSs, we carried out protein-protein interactions (PPI) among 24 genes of ADAMTSs through STRING website and visualized them by Cytoscape software (Figure 2(a)). Through the analysis, we found that there is a connection between each member of the ADAMTSs and the other 23 members (Figure 2(b)). In order to further explore the relationship between members of the ADAMTSs, we analyzed the coexpression of genes by “Corrplot” package. We found that there is a strong correlation among ADAMTSs (Figure 2(c)), in which the Pearson correlation coefficients between ADAMTS12 and ADAMTS14 and ADAMTS2 are 0.752 (Figure 2(d)) and 0.524 (Figure 2(e)), respectively.

### 3.4. Functional Analysis of ADAMTSs

Through the analysis of the classical pathway of the ADAMTSs, we found that the pathway closely related to the ADAMTSs is mainly apoptosis, cell cycle, DNA damage response, EMT, hormone AR, hormone ER, PI3K/AKT, RTK, and TSC/mTOR (Figures S2A and S2B). ADAMTSs can cause significant inhibition of cell cycle and activation of EMT. Then, we carried out the drug sensitivity analysis and found that the ADAMTS is closely related to the sensitivity of many kinds of drugs (Figure S2C).

### 3.5. ADAMTSs Are Closely Related to Poor Prognosis

**Figure 3:**
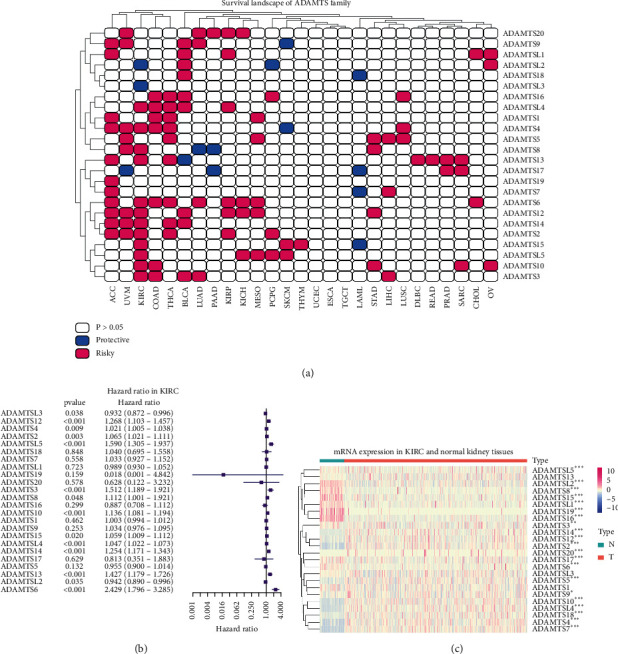
Effect of ADAMTSs on survival of cancer patients: (a) analysis of the influence of ADAMTSs on patient survival. Red represents risk factors and blue represents protective factors. (b) Hazard ratio analysis of ADAMTSs in KIRC. (c) The difference of ADAMTS mRNA expression between renal cell carcinoma and normal renal tissue.

We analyzed the influence of ADAMTSs on prognosis in different types of cancer by B software. The results showed that ADAMTS was a risk factor in most cancers (Figure 3(a); Tables S6 and S7). In order to further explore the effect of ADAMTSs on the prognosis of KIRC, univariate Cox regression analysis was used to analyze the expression of ADAMTSs in TCGA database. The results showed that the high expression of ADAMTSL2 and ADAMTSL3 was related to better prognosis, and the high expression of ADAMTSL4, ADAMTS4, ADAMTS8, ADAMTS13, ADAMTS6, ADAMTS12, ADAMTS14, ADAMTS2, ADAMTS15, ADAMTSL5, ADAMTS10, and ADAMTS3 was related to poor prognosis (Figure 3(b)). Then, we further analyzed the expression of ADAMTSs in renal clear cell carcinoma (Figure 3(c); Table S8). We found that ADAMTSL5, ADAMTSL1, ADAMTS19, ADAMTS3, ADAMTS8, ADAMTS16, ADAMTS15, ADAMTS17, ADAMTSL2, and ADAMTS6 were low expressed in ccRCC and ADAMTS12, ADAMTS4, ADAMTS2, ADAMTS18, ADAMTS7, ADAMTS20, ADAMTS10, ADAMTS9, ADAMTSL4, ADAMTS14, ADAMTS5, and ADAMTS13 were highly expressed in ccRCC.

### 3.6. Establishment and Verification of the Prognostic Model Based on ADAMTS

**Figure 4:**
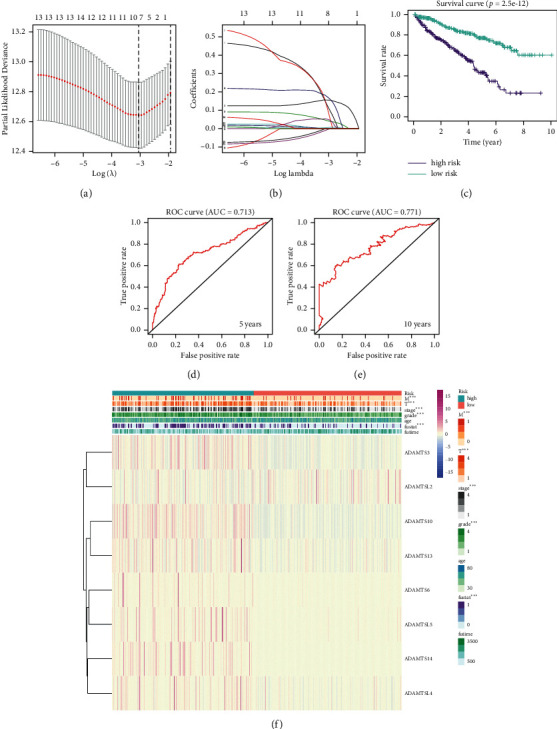
The establishment of prognostic model based on ADAMTSs in KIRC. (a) Partial likelihood deviance was plotted against log (lambda). (b) The lasso coefficient profiles of ADAMTSs in KIRC. (c) Patients were grouped based on risk scores, and the Kaplan–Meier survival curve showed the OS rate of KIRC patients in both the high-risk and the low-risk groups. (d, e) ROC curve analysis showed that the new survival model was efficient in predicting prognosis, and the AUC values for 5-year survival and 10-year survival were 0.713 and 0.771, respectively. (f) The relationship between risk score and clinical case characteristics.

Firstly, ADAMTS was selected as the survival-related ADAMTS, according to *P* < 0.05. Then, by using the lasso regression model, we determined the strongest prognostic markers, and based on the minimum criterion, eight genes (ADAMTS3, ADAMTSL2, ADAMTS10, ADAMTS13, ADAMTS6, ADAMTSL5, ADAMTS14, and ADAMTSL4) were selected according to the analysis results to establish a risk signature model (Figures 4(a) and 4(b)). Then, according to the median risk score, we divided patients with renal clear cell carcinoma into low-risk group and high-risk group. Kaplan–Meier survival curve analysis showed that the survival rate of patients in the low-risk group was significantly better than that in the high-risk group (Figure 4(c)). Furthermore, in order to analyze the predictive effect of the new prognostic model on the prognosis of patients with KIRC, we also carried out ROC curve analysis. The AUC score of 5-year survival rate was 0.713 and the AUC score of 10-year survival rate was 0.771(Figures 4(d) and 4(e)). In order to better explore the relationship between ADAMTSs and KIRC, we analyzed the correlation between risk scores based on eight ADAMTSs and the clinicopathological characteristics of high-risk and low-risk patients with KIRC in TCGA database. We observed that the risk score was strongly correlated with the clinicopathological features of patients with high-risk and low-risk clear cell renal cell carcinoma, such as *T*, *M*, tumor grade, tumor stage, and fustat (Figure 4(f)). Then, we validated the model in the GSE22541 dataset of the GEO database, and the results show that the prognosis of the low-risk group is significantly better than that of the high-risk group based on this model, which is consistent with the data from the TCGA database (Figure S3).

**Figure 5:**
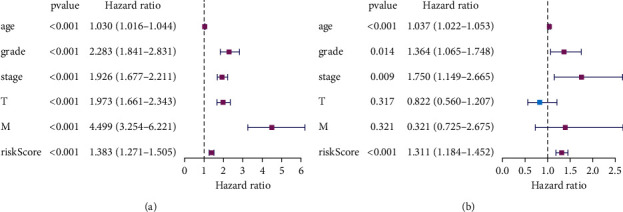
Univariate and multivariate Cox regression analyses. (a) Univariate Cox regression analysis showed that the clinicopathological parameters including age, grade, stage, *T, M*, and risk score of the prognostic model were correlated with OS in patients with renal cell carcinoma. (b) Multivariate Cox regression analysis showed that age, grade, stage, and risk score were independent risk factors affecting the prognosis of patients with clear cell renal cell carcinoma.

Univariate Cox regression analysis showed that age, grade, tumor stage, *T*, *M,* and risk score were associated with OS in patients with renal cell carcinoma (Figure 5(a); Table S9). Multivariate Cox regression analysis showed that risk score, age, grade, and stage were independent risk factors affecting the prognosis of patients with ccRCC (Figure 5(b); Table S10).

### 3.7. Pathway Analysis of Key Genes in Prognostic Model Based on ADAMTS

To further explore the related pathways of key genes in the ADAMTS-based survival model, we analyzed the related pathways of key genes through GSEA website. The high expression of ADAMTSL2 could promote the activation of ECM receptor interaction, MAPK signaling pathway, Wnt signaling pathway, and pathways in cancer, while the low expression of ADAMTSL2 could inhibit the biosynthesis of unsaturated fatty acid (Figure S4A). The high expression of ADAMTS4 could significantly promote the activation of ECM receptor interaction, MAPK signaling pathway, notch signaling pathway, TGF-beta signaling pathway, and VEGF signaling pathway (Figure S4B). The low expression of ADAMTS10 could inhibit the biosynthesis of unsaturated fatty acid, citrate cycle (TCA cycle), glycolysis gluconeogenesis, propanoate metabolism, and pyruvate metabolism (Figure S4C). Similarly, the low expression of ADAMTS14 could conspicuously inhibit the TCA cycle, fatty acid metabolism, histidine metabolism, propanoate metabolism, and pyruvate metabolism (Figure S4D).

## 4. Discussion

With the aging of the population, the deterioration of the environment, and the increase of the population base, cancer begins to appear in the public view more and more frequently, and there are more and more new cancer cases and deaths due to cancer every year. According to the American Cancer Society, there will be 1806590 new cancer cases in 2020 and 606520 people will die of cancer [22]. Although the cancer mortality rate has been declining since 1991 [22], the number of deaths from cancer is still large. So, cancer is still an important obstacle to human health.

More and more reports are confirming the interaction between ADAMTSs and tumor. ADAMTS1 plays an antiangiogenic role in liver metastases by regulating thrombospondin-1 (TSP1) [23]. ADAMTS1 is low expressed in breast carcinoma and plays an inhibitory role in breast cancer [9]. ADAMTS15 is low expressed in colorectal carcinomas and inhibits tumor growth and invasion [24]. ADAMTSs may play different roles in different tumors, so it is necessary to analyze the mutation of ADAMTSs across cancer types. Through CNV analysis and SNV analysis, we found that ADAMTSs have a wide range of mutations in 32 kinds of tumors, which is undoubtedly a key signal that ADAMTSs may play an important role in the occurrence and development of tumors. ADAMTSs can also regulate tumors through epigenetic changes. ADAMTS1 shows high frequency promoter methylation in lung and pancreatic cancers [25, 26]; ADAMTS5 and ADAMTS1 also show high frequency methylation in colorectal cancer [27, 28]. In renal cell carcinoma, melatonin-triggered posttranscriptional and posttranslational modification of ADAMTS1 synergistically inhibits renal cell carcinoma [12]. Our analysis results also show the importance of methylation for ADAMTSs. Many kinds of ADAMTSs show high levels of methylation in many kinds of tumors, especially ADAMTS20, ADAMTS8, ADAMTS10, and ADAMTS3. The correlation analysis between methylation and gene expression level shows that methylation is negatively correlated with gene expression, which means that the main role of ADAMTS methylation is to silence ADAMTS gene, which leads to the decrease of ADAMTS expression. Moreover, the hypermethylation of ADAMTSs represents a higher risk of survival, which makes the study of ADAMTSs more clinically significant and inspires us to study the relationship between ADAMTSs and the prognosis of tumor patients.

The differential expression of the gene in tumor tissue and normal tissue often indicates that the gene may play an important role as a proto-oncogene or tumor suppressor gene in the occurrence and development of tumor. ADAMTS is significantly differentially expressed in many kinds of tumors, such as KIRC, KICH, KIRP, LUAD, and LUSC, especially ADAMTS20 and ADAMTS14 are highly expressed in most tumors, and the results of TIMER website are consistent with our analysis results. This may mean that ADAMTS20 and ADAMTS14 deserve more attention from oncology researchers. The correlation analysis of the ADAMTSs shows that there is a strong correlation between the members of the ADAMTSs, which may mean that the members of the ADAMTSs do not play a single role, but influence each other, or even have a synergistic effect. In order to explore the main pathways through which the ADAMTSs play a role, we have analyzed the classical pathways. The main pathways involved in the ADAMTSs are apoptosis, cell cycle, DNA damage response, EMT, and so on. This provides a direction for our future research.

What kind of influence the gene changes in tumor have on the prognosis of tumor patients is often the most concerned part of clinicians and patients. Our prognostic analysis in a variety of tumors found that the ADAMTSs had an impact on the prognosis of patients with 24 kinds of tumors, especially in patients with ACC, UVM, KIRC, COAD, THCA, and so on. Among the 24 kinds of tumor patients, the ADAMTS has the most significant impact on the prognosis of KIRC patients, as many as 14 ADAMTS family members can affect the prognosis of KIRC patients. This prompted us to establish a KIRC prognosis prediction model based on ADAMTSs.

There are several grouped variable selection methods such as elastic net, lasso, and net [29]. Since there are 24 genes in the ADAMTS family, the number of independent variables was so large that the results were bound to overfit. As Pak et al. said, the lasso helps to reduce the choice of variables [29]. Therefore, we adopted lasso regression instead of ridge regression to reduce unnecessary genes that have overfitting effects on the predicted results, so as to reduce the number of independent variables.

The lasso regression model was used to determine the most reliable prognostic indicators and selected 8 genes according to the analysis results. Survival analysis showed that the prognosis of the low-risk group was significantly better than that of the high-risk group. We used ROC curve to evaluate the accuracy of the prognostic analysis model in predicting 5-year and 10-year survival rates, with AUC values of 0.713 and 0.771, respectively, indicating that our prognostic analysis model for renal cell carcinoma is reliable. Further analysis showed that the risk score based on the ADAMTSs was closely related to the clinicopathological features, and the higher risk score represented the higher *T*, *M*, tumor grade, tumor stage, and fustat. Multivariate Cox regression analysis showed that, like age, stage, and grade of renal cell carcinoma, the risk score of the prognostic model was also independently correlated with the prognosis of ccRCC, which was an important index to determine the prognosis, which further proved the effectiveness of the prognostic analysis model based on ADAMTSs.

In order to explore the pathways in which these eight key genes play a role, we carried out pathway analysis. During this period, four genes were excluded, and the criteria for our analysis were that the gene is highly expressed and belongs to a risk factor, or that the gene is low expressed and belongs to a protective factor. Although ADAMTSL5 is a risk factor for the prognosis of renal cell carcinoma, the change of its expression in KIRC has no significance in the analysis of gene expression changes across cancer types, so we do not analyze its pathway, and similarly, we do not analyze the pathway of ADAMTS3, ADAMTS6, and ADAMTS13. Most of the pathways involved in these key genes are very classical signal pathways, such as the MAPK signal pathway, WNT signal pathway, and fatty acid metabolism. According to previous studies, WNT signaling pathway and fatty acid metabolism are closely related to clear cell renal cell carcinoma [30–32], which further shows the important value of ADAMTSs in clear cell renal cell carcinoma, which is worthy of our in-depth study.

However, there are still some limitations in this study. We did not analyze the pathway of ADAMTSL5, ADAMTS3, ADAMTS6, and ADAMTS13 among the 8 genes used to establish the prognosis model because there was no significant difference in the expression of ADAMTSL5, ADAMTS3, ADAMTS6, and ADAMTS13 between ccRCC and normal renal tissue, or although they are risk factors, compared with normal renal tissue, their expression levels in ccRCC are lower, so there is no need for pathway analysis.

In conclusion, ADAMTS had a wide range of mutations and differential expression across cancer types and was closely related to the prognosis of many cancers. The prognostic model based on ADAMTSs could predict the prognosis of patients with renal clear cell carcinoma. Multivariate Cox regression analysis showed that risk score was an independent prognostic factor for ccRCC. Our study suggests that ADAMTS is a potential prognostic biomarker and therapeutic target for ccRCC.

## Data Availability

The data supporting the results of this study are available from the corresponding author upon request.

## Supplementary Materials

Supplementary Materials Figure S1: alternations of gene expression of ADAMTSs across cancer types. A: alternations of gene expression of ADAMTSs across cancer types. B-C: changes of gene expression of ADAMTS20 and ADAMTS14 in different types of cancer. E-F: the protein expression level of ADAMTS20 in KIRC and normal renal tissue. Figure S2: classical pathway analysis and drug sensitivity analysis of ADAMTSs. A-B: classical pathway analysis of ADAMTSs. C: drug sensitivity analysis of ADAMTSs. Figure S3: verification of the prognostic model based on ADAMTS (GSE22541). Figure S4: gene set enrichment analysis in KIRC. A: ADAMTSL2; B: ADAMTSL4; C: ADAMTS10; D: ADAMTS14. Table S1: CNV amplification frequency of ADAMTSs across cancer types. Table S2: CNV deletion frequency of ADAMTSs across cancer types. Table S3: SNV frequency of ADAMTSs across cancer types. Table S4: Log FCs of the expression of ADAMTSs across cancer types. Table S5: *P* values of the expression of ADAMTSs across cancer types. Table S6: HRs of ADAMTSs across cancer types. Table S7: *P* values of the HRs of ADAMTSs across cancer types. Table S8: the differential expression of ADAMTSs in KIRC and normal renal tissues. Table S9: univariate Cox regression analysis. Table S10: multivariate Cox regression analysis. .
