# Supplementary material for: A New Survival Model Based on ADAMTSs for Prognostic Prediction in Clear Cell Renal Cell Carcinoma
Source: J Oncol. 2021 Sep 23;2021:2606213. doi: 10.1155/2021/2606213 (PMC8486512; doi:10.1155/2021/2606213)
Supplement: Supplementary Materials — Figure S1: alternations of gene expression of ADAMTSs across cancer types. A: alternations of gene expression of ADAMTSs across cancer types. B-C: changes of gene expression of ADAMTS20 and ADAMTS14 in different types of cancer. E-F: the protein expression level of ADAMTS20 in KIRC and normal renal tissue. Figure S2: classical pathway analysis and drug sensitivity analysis of ADAMTSs. A-B: classical pathway analysis of ADAMTSs. C: drug sensitivity analysis of ADAMTSs. Figure S3: verification of the prognostic model based on ADAMTS (GSE22541). Figure S4: gene set enrichment analysis in KIRC. A: ADAMTSL2; B: ADAMTSL4; C: ADAMTS10; D: ADAMTS14. Table S1: CNV amplification frequency of ADAMTSs across cancer types. Table S2: CNV deletion frequency of ADAMTSs across cancer types. Table S3: SNV frequency of ADAMTSs across cancer types. Table S4: Log FCs of the expression of ADAMTSs across cancer types. Table S5: P values of the expression of ADAMTSs across cancer types. Table S6: HRs of ADAMTSs across cancer types. Table S7: P values of the HRs of ADAMTSs across cancer types. Table S8: the differential expression of ADAMTSs in KIRC and normal renal tissues. Table S9: univariate Cox regression analysis. Table S10: multivariate Cox regression analysis. . [file 2606213.f1.zip › 2606213.f1/FIG S2 (1).pdf]

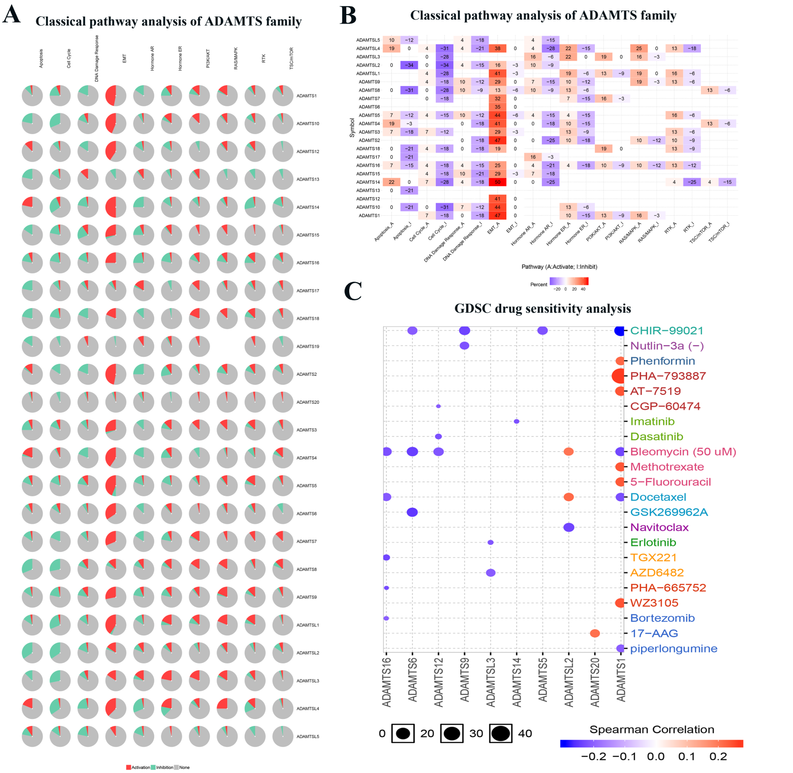
A
Classical pathway analysis of ADAMTS family
Apoptosis
Cell Cycle
DNA Damage Response
EMT
Hormone AR
Hormone ER
PI3K/AKT
RAS/MAPK
RTK
TSC/mTOR
ADAMTS1
ADAMTS10
ADAMTS12
ADAMTS13
ADAMTS14
ADAMTS15
ADAMTS16
ADAMTS17
ADAMTS18
ADAMTS19
ADAMTS2
ADAMTS20
ADAMTS3
ADAMTS4
ADAMTS5
ADAMTS6
ADAMTS7
ADAMTS8
ADAMTS9
ADAMTSL1
ADAMTSL2
ADAMTSL3
ADAMTSL4
ADAMTSL5
Activation
Inhibition
None
B
Classical pathway analysis of ADAMTS family
Symbol
Pathway (A:Activate; I:Inhibit)
Percent
C
GDSC drug sensitivity analysis
CHIR−99021
Nutlin−3a (−)
Phenformin
PHA−793887
AT−7519
CGP−60474
Imatinib
Dasatinib
Bleomycin (50 uM)
Methotrexate
5−Fluorouracil
Docetaxel
GSK269962A
Navitoclax
Erlotinib
TGX221
AZD6482
PHA−665752
WZ3105
Bortezomib
17−AAG
piperlongumine
ADAMTS16
ADAMTS6
ADAMTS12
ADAMTS9
ADAMTSL3
ADAMTS14
ADAMTS5
ADAMTSL2
ADAMTS20
ADAMTS1
0
20
30
40
Spearman Correlation
−0.2
−0.1
0.0
0.1
0.2
