# Supplementary material for: A New Survival Model Based on ADAMTSs for Prognostic Prediction in Clear Cell Renal Cell Carcinoma
Source: J Oncol. 2021 Sep 23;2021:2606213. doi: 10.1155/2021/2606213 (PMC8486512; doi:10.1155/2021/2606213)
Supplement: Supplementary Materials — Figure S1: alternations of gene expression of ADAMTSs across cancer types. A: alternations of gene expression of ADAMTSs across cancer types. B-C: changes of gene expression of ADAMTS20 and ADAMTS14 in different types of cancer. E-F: the protein expression level of ADAMTS20 in KIRC and normal renal tissue. Figure S2: classical pathway analysis and drug sensitivity analysis of ADAMTSs. A-B: classical pathway analysis of ADAMTSs. C: drug sensitivity analysis of ADAMTSs. Figure S3: verification of the prognostic model based on ADAMTS (GSE22541). Figure S4: gene set enrichment analysis in KIRC. A: ADAMTSL2; B: ADAMTSL4; C: ADAMTS10; D: ADAMTS14. Table S1: CNV amplification frequency of ADAMTSs across cancer types. Table S2: CNV deletion frequency of ADAMTSs across cancer types. Table S3: SNV frequency of ADAMTSs across cancer types. Table S4: Log FCs of the expression of ADAMTSs across cancer types. Table S5: P values of the expression of ADAMTSs across cancer types. Table S6: HRs of ADAMTSs across cancer types. Table S7: P values of the HRs of ADAMTSs across cancer types. Table S8: the differential expression of ADAMTSs in KIRC and normal renal tissues. Table S9: univariate Cox regression analysis. Table S10: multivariate Cox regression analysis. . [file 2606213.f1.zip › 2606213.f1/Supplementary files.pdf]

Table S1

	ACC	BLCA	BRCA	CESC	CHOL	COAD	DLBC
ADAMTS1	0.188889	0.103865	0.058107	0.040816	0	0.014028	0.068966
ADAMTS2	0.577778	0.012077	0.073102	0.040816	0.121212	0.022044	0.034483
ADAMTS3	0.3	0.062802	0.071228	0.032653	0	0.01002	0
ADAMTS4	0.088889	0.386473	0.584817	0.342857	0.484848	0.11022	0.275862
ADAMTS5	0.188889	0.111111	0.062793	0.040816	0	0.014028	0.068966
ADAMTS6	0.544444	0.016908	0.043112	0.020408	0.060606	0.016032	0.034483
ADAMTS7	0.077778	0.019324	0.048735	0.106122	0.060606	0.014028	0.034483
ADAMTS8	0.033333	0.033816	0.021556	0.004082	0	0.016032	0.172414
ADAMTS9	0.066667	0.070048	0.015933	0.012245	0	0.032064	0.103448
ADAMTS10	0.433333	0.036232	0.049672	0.053061	0	0.034068	0
ADAMTS12	0.566667	0.304348	0.164011	0.359184	0.151515	0.098196	0.068966
ADAMTS13	0.244444	0.048309	0.054358	0.146939	0.030303	0.058116	0.068966
ADAMTS14	0.166667	0.02657	0.037488	0.004082	0.121212	0.012024	0
ADAMTS15	0.033333	0.033816	0.020619	0.004082	0	0.014028	0.172414
ADAMTS16	0.533333	0.297101	0.1612	0.367347	0.151515	0.076152	0.068966
ADAMTS17	0.088889	0.02657	0.085286	0.126531	0.060606	0.018036	0.034483
ADAMTS18	0.377778	0.062802	0.029991	0.073469	0.030303	0.07014	0.034483
ADAMTS19	0.555556	0.009662	0.049672	0.032653	0.151515	0.016032	0.068966
ADAMTS20	0.655556	0.060386	0.061856	0.053061	0.060606	0.09018	0.034483
ADAMTSL1	0.155556	0.070048	0.072165	0.097959	0	0.096192	0.068966
ADAMTSL2	0.244444	0.048309	0.055295	0.146939	0.030303	0.058116	0.068966
ADAMTSL3	0.077778	0.028986	0.059981	0.106122	0.060606	0.016032	0.034483
ADAMTSL4	0.111111	0.306763	0.558575	0.338776	0.484848	0.106212	0.206897
ADAMTSL5	0.411111	0.02657	0.014995	0.02449	0.030303	0.028056	0

ESCA	GBM	HNSC	KICH	KIRC	KIRP	LGG	LIHC	LUAD
0.041667	0.057325	0.017241	0.121212	0.033956	0.046053	0.02439	0.015831	0.055985
0.052083	0.012739	0.017241	0.348485	0.370119	0.108553	0.011257	0.189974	0.086873
0.104167	0.017516	0.068966	0.409091	0.010187	0.006579	0.009381	0.013193	0.023166
0.208333	0.063694	0.146552	0	0.061121	0.032895	0.0394	0.60686	0.391892
0.041667	0.057325	0.017241	0.121212	0.033956	0.046053	0.02439	0.015831	0.055985
0.020833	0.023885	0.008621	0.287879	0.120543	0.059211	0.009381	0.094987	0.044402
0.052083	0.011146	0.043103	0.409091	0.006791	0.006579	0.018762	0.047493	0.025097
0.052083	0.019108	0.051724	0.242424	0.011885	0.029605	0.06379	0.029024	0.088803
0	0.031847	0.008621	0.212121	0.010187	0.167763	0.009381	0.031662	0.021236
0	0.299363	0.008621	0.318182	0.01528	0.009868	0.050657	0.021108	0.011583
0.416667	0.033439	0.224138	0.257576	0.140917	0.082237	0.022514	0.189974	0.391892
0.197917	0.058917	0.12931	0.257576	0.013582	0.003289	0.033771	0.031662	0.017375
0.03125	0.003185	0.008621	0.045455	0.005093	0.032895	0.005629	0.026385	0.040541
0.052083	0.019108	0.051724	0.242424	0.011885	0.029605	0.06379	0.029024	0.088803
0.46875	0.028662	0.258621	0.212121	0.149406	0.092105	0.016886	0.211082	0.438224
0.072917	0.019108	0.103448	0.363636	0.006791	0.013158	0.033771	0.060686	0.044402
0.083333	0.022293	0.060345	0.257576	0.039049	0.384868	0.011257	0.010554	0.046332
0	0.015924	0	0.272727	0.325976	0.085526	0.007505	0.14248	0.032819
0.072917	0.047771	0.034483	0.348485	0.04584	0.325658	0.009381	0.031662	0.081081
0.0625	0.015924	0.086207	0.227273	0.013582	0.013158	0.011257	0.023747	0.025097
0.197917	0.058917	0.12931	0.257576	0.013582	0.003289	0.033771	0.034301	0.019305
0.083333	0.014331	0.060345	0.378788	0.005093	0.006579	0.020638	0.05277	0.027027
0.1875	0.078025	0.12931	0.015152	0.057725	0.029605	0.041276	0.577836	0.457529
0.020833	0.278662	0.008621	0.318182	0.011885	0.009868	0.060038	0.031662	0.009653

LUSC	MESO	OV	PAAD	PCPG	PRAD	READ	SARC	SKCM
0.032443	0.034884	0.104928	0	0	0.016227	0.048193	0.158333	0.057203
0.013359	0.081395	0.154213	0.020134	0.02	0.014199	0.084337	0.116667	0.057203
0.061069	0	0.120827	0	0.02	0.018256	0.060241	0.116667	0.042373
0.246183	0.139535	0.434022	0.194631	0.113333	0.024341	0.204819	0.316667	0.332627
0.032443	0.034884	0.104928	0	0	0.016227	0.048193	0.158333	0.059322
0.003817	0.046512	0.020668	0	0.026667	0.002028	0.048193	0.125	0.044492
0.066794	0.116279	0.112878	0.040268	0.06	0.004057	0.012048	0.141667	0.118644
0.051527	0.046512	0.195548	0.020134	0.006667	0.014199	0.012048	0.05	0.006356
0.003817	0.046512	0.104928	0.006711	0.013333	0.010142	0.048193	0.116667	0.057203
0.051527	0.081395	0.17965	0.006711	0.066667	0.004057	0.036145	0.216667	0.029661
0.522901	0.209302	0.375199	0.026846	0.06	0.020284	0.156627	0.316667	0.129237
0.053435	0.011628	0.055644	0	0	0.052738	0.036145	0.066667	0.019068
0.024809	0	0.128776	0.013423	0.026667	0.01217	0.024096	0.008333	0
0.051527	0.046512	0.193959	0.020134	0.006667	0.014199	0.012048	0.066667	0.006356
0.545802	0.209302	0.435612	0.04698	0.046667	0.028398	0.084337	0.25	0.137712
0.148855	0.127907	0.214626	0.04698	0.066667	0.006085	0.060241	0.233333	0.125
0.030534	0.011628	0.038156	0	0.02	0.006085	0.120482	0.058333	0.029661
0	0.05814	0.065183	0	0.033333	0.01217	0.060241	0.108333	0.03178
0.070611	0.034884	0.227345	0.006711	0.026667	0.006085	0.072289	0.066667	0.036017
0.043893	0	0.163752	0.006711	0.013333	0.010142	0.108434	0.133333	0.016949
0.053435	0.011628	0.057234	0	0	0.05071	0.036145	0.066667	0.019068
0.101145	0.139535	0.143084	0.040268	0.053333	0.008114	0.012048	0.158333	0.116525
0.248092	0.151163	0.510334	0.201342	0.146667	0.042596	0.228916	0.35	0.313559
0.028626	0.069767	0.011129	0	0.073333	0.004057	0.048193	0.116667	0.029661

STAD	TGCT	THCA	THYM	UCEC	UCS	UVM
0.00905	0.525641	0	0.033333	0.027322	0.178571	0.1875
0.0181	0	0.021526	0	0.04918	0.089286	0.0125
0.036199	0.038462	0.001957	0	0.021858	0.035714	0.05
0.124434	0.147436	0.054795	0.066667	0.342441	0.482143	0.0875
0.00905	0.525641	0	0.033333	0.027322	0.196429	0.1875
0.022624	0	0.013699	0	0.021858	0.053571	0
0.040724	0.038462	0	0.033333	0.014572	0.089286	0.0125
0.049774	0	0	0	0.032787	0.107143	0.075
0.027149	0.076923	0	0	0.030965	0.089286	0
0.004525	0.019231	0	0	0.067395	0.035714	0.0125
0.156109	0	0.027397	0.033333	0.100182	0.357143	0
0.065611	0.012821	0.003914	0	0.014572	0.017857	0.05
0.058824	0.025641	0	0	0.134791	0.160714	0
0.049774	0	0	0	0.032787	0.107143	0.075
0.142534	0.012821	0.029354	0.033333	0.098361	0.357143	0
0.104072	0.051282	0	0.033333	0.03643	0.142857	0.0125
0.038462	0.012821	0.005871	0	0.007286	0	0
0.015837	0	0.019569	0	0.014572	0.071429	0
0.036199	0.205128	0.007828	0	0.047359	0.267857	0
0.033937	0.019231	0	0	0.054645	0.035714	0.0625
0.065611	0.012821	0.003914	0	0.007286	0.017857	0.05
0.058824	0.038462	0	0.033333	0.016393	0.125	0.0125
0.149321	0.134615	0.054795	0.066667	0.380692	0.571429	0.075
0.013575	0.00641	0	0	0.003643	0.017857	0

Table S2

	ACC	BLCA	BRCA	CESC	CHOL	COAD	DLBC	ESCA
ADAMTS1	0.06667	0.02657	0.05904	0.04898	0.18182	0.10621	0	0.13542
ADAMTS2	0	0.16184	0.05342	0.12245	0	0.04409	0.06897	0.1875
ADAMTS3	0.02222	0.02899	0.04405	0.0898	0.12121	0.05611	0.06897	0.0625
ADAMTS4	0.07778	0	0	0	0	0	0	0
ADAMTS5	0.06667	0.02415	0.05904	0.04898	0.18182	0.10621	0	0.13542
ADAMTS6	0.01111	0.15942	0.11528	0.11837	0.0303	0.07214	0	0.20833
ADAMTS7	0.1	0.05072	0.04873	0.04082	0	0.13226	0.06897	0.03125
ADAMTS8	0.2	0.13527	0.26054	0.35102	0.12121	0.02605	0	0.27083
ADAMTS9	0.12222	0.0942	0.11528	0.33878	0.51515	0.03607	0	0.39583
ADAMTS10	0.02222	0.05797	0.04217	0.1102	0	0.01804	0	0.04167
ADAMTS12	0.01111	0.00966	0.03093	0.01224	0	0.02405	0.03448	0.02083
ADAMTS13	0.01111	0.17391	0.07591	0.01633	0.24242	0.02004	0	0.07292
ADAMTS14	0	0.10386	0.05717	0.08571	0.06061	0.0521	0.06897	0.05208
ADAMTS15	0.2	0.13527	0.26148	0.35102	0.12121	0.02806	0	0.27083
ADAMTS16	0.1	0.02174	0.02905	0.02857	0.0303	0.03006	0	0.03125
ADAMTS17	0.11111	0.04831	0.06842	0.03265	0	0.12826	0.06897	0.03125
ADAMTS18	0.07778	0.09903	0.34114	0.06939	0.09091	0.01804	0.03448	0.02083
ADAMTS19	0.01111	0.10628	0.07591	0.09796	0	0.08016	0.10345	0.17708
ADAMTS20	0.01111	0.02415	0.02156	0.00408	0	0.01403	0	0.03125
ADAMTSL1	0.18889	0.22947	0.10684	0.06939	0.24242	0.0501	0.03448	0.19792
ADAMTSL2	0.01111	0.17391	0.07685	0.01633	0.24242	0.02004	0	0.07292
ADAMTSL3	0.11111	0.04106	0.0478	0.04082	0	0.12425	0.06897	0.02083
ADAMTSL4	0.08889	0	0	0	0	0.002	0	0
ADAMTSL5	0.02222	0.08937	0.1059	0.19184	0	0.04208	0	0.10417

GBM	HNSC	KICH	KIRC	KIRP	LGG	LIHC	LUAD	LUSC
0.03822	0.08621	0.30303	0.03396	0.09539	0.02064	0.11873	0.06757	0.07634
0.0414	0.16379	0.13636	0.00849	0.00658	0.03377	0.01583	0.05405	0.18702
0.0207	0.06897	0	0.01528	0.04276	0.06004	0.20053	0.01544	0.0458
0.00159	0	0.57576	0.01868	0.03618	0	0	0	0
0.03822	0.08621	0.30303	0.03735	0.09539	0.02064	0.11873	0.06757	0.07443
0.01752	0.15517	0.09091	0.00509	0.01645	0.01313	0.01847	0.07143	0.15267
0.07643	0.03448	0	0.00679	0.02303	0.02439	0.05277	0.03668	0.01145
0.02866	0.11207	0.13636	0.00679	0.04276	0.01126	0.1029	0.07143	0.06679
0.02866	0.26724	0.06061	0.37351	0.03947	0.04128	0.06069	0.05212	0.1813
0.00796	0.03448	0	0.00679	0.01316	0	0.08707	0.10039	0.05344
0.02707	0	0.09091	0.00509	0.00329	0.03002	0.00264	0.00965	0.01145
0.07166	0.01724	0.06061	0.05603	0.07566	0.02251	0.09763	0.04633	0.05534
0.69745	0.05172	0.63636	0.05093	0.03289	0.09944	0.06069	0.01158	0.08015
0.02866	0.11207	0.13636	0.00849	0.04276	0.01126	0.1029	0.0695	0.06679
0.04936	0.00862	0.16667	0.00509	0.00987	0.0469	0.01319	0.00772	0.01527
0.07325	0.00862	0	0.01358	0.02961	0.02814	0.08179	0.03861	0.01336
0.08917	0.08621	0.06061	0.01528	0.01645	0.01689	0.25066	0.05212	0.08206
0.02866	0.14655	0.09091	0.00849	0.00987	0.03189	0.02902	0.05985	0.15267
0.07803	0	0	0	0	0.0469	0.01055	0.02124	0.00954
0.48248	0.24138	0.07576	0.05433	0.08553	0.17261	0.16095	0.13514	0.16221
0.07325	0.01724	0.06061	0.05603	0.07566	0.02251	0.09763	0.04826	0.05534
0.07803	0.02586	0	0.01188	0.02303	0.02251	0.05805	0.02896	0.01336
0	0	0.56061	0.01868	0.03289	0	0	0	0
0.01115	0.10345	0	0.00849	0.02303	0.00563	0.09763	0.11776	0.07252

MESO	OV	PAAD	PCPG	PRAD	READ	SARC	SKCM	STAD
0.04651	0.15421	0.0604	0.13333	0.0284	0.19277	0.01667	0.05932	0.08597
0.02326	0.19237	0.02013	0.03333	0.00609	0.12048	0.08333	0.12712	0.05656
0.10465	0.26391	0	0.04	0.00203	0.07229	0.04167	0.06356	0.02489
0	0.00795	0	0.02667	0.02637	0	0.00833	0.00424	0
0.04651	0.15898	0.0604	0.15333	0.0284	0.19277	0.01667	0.05932	0.08597
0.03488	0.45787	0.04027	0.02667	0.10548	0.18072	0.03333	0.09958	0.09502
0.01163	0.23211	0.02685	0.00667	0.0142	0.16867	0.05	0.02119	0.02941
0.02326	0.17329	0.00671	0.18667	0.02231	0.03614	0.20833	0.31356	0.04072
0.11628	0.1097	0.03356	0.20667	0.02231	0.03614	0.15	0.03178	0.02262
0.01163	0.23529	0.01342	0	0.01014	0.06024	0	0.02754	0.06787
0	0.03657	0	0.04	0.01623	0.03614	0.025	0.0572	0.02941
0.0814	0.33068	0.02685	0.03333	0.00203	0.04819	0.125	0.2161	0.01584
0.04651	0.09698	0.02685	0	0.03043	0.03614	0.26667	0.30932	0.02715
0.02326	0.17647	0.00671	0.18667	0.02028	0.03614	0.20833	0.31356	0.04525
0	0.04293	0	0.02	0.01623	0.04819	0.075	0.08263	0.02489
0.01163	0.17965	0.02013	0.01333	0.01826	0.18072	0.04167	0.0339	0.02262
0.06977	0.55803	0.03356	0.03333	0.20892	0.03614	0.18333	0.15042	0.05656
0.05814	0.27663	0.01342	0.06	0.03245	0.16867	0.075	0.12712	0.08371
0.02326	0.06518	0.02013	0.04	0.01014	0.03614	0.05	0.0678	0.03394
0.11628	0.27027	0.12752	0.03333	0.02231	0.08434	0.23333	0.36864	0.11312
0.0814	0.3275	0.02685	0.03333	0.00203	0.04819	0.125	0.2161	0.01584
0.01163	0.19873	0.03356	0.00667	0.01217	0.16867	0.03333	0.01907	0.02036
0.01163	0.00159	0	0.01333	0	0	0.00833	0.00212	0
0.04651	0.59618	0.01342	0	0.02028	0.07229	0.10833	0.06144	0.07014

TGCT	THCA	THYM	UCEC	UCS	UVM
0	0.00196	0.03333	0.02004	0.10714	0.0125
0.07051	0.00196	0	0.04007	0.21429	0.0625
0.02564	0	0	0.04918	0.30357	0.0625
0	0	0	0.00182	0	0
0	0.00196	0.03333	0.02186	0.10714	0.0125
0.02564	0	0	0.10383	0.28571	0.05
0.00641	0.01174	0	0.08197	0.23214	0.05
0.27564	0.0137	0	0.08743	0.25	0.0375
0	0	0.1	0.06557	0.35714	0.5125
0.01282	0.01174	0	0.1184	0.46429	0.025
0.03205	0	0	0.02004	0.01786	0.0125
0.00641	0.03914	0.03333	0.11658	0.41071	0.0375
0.01282	0.00978	0	0.04918	0.08929	0
0.27564	0.0137	0	0.08743	0.25	0.0375
0.03846	0.00783	0	0.02004	0.01786	0.025
0.01282	0.01174	0.03333	0.07104	0.17857	0.0625
0.04487	0.00978	0.06667	0.20765	0.35714	0.25
0.04487	0	0	0.06375	0.08929	0.0625
0.01282	0	0	0.01275	0.07143	0.025
0.03205	0.01957	0.1	0.06011	0.26786	0.0375
0.00641	0.03914	0.03333	0.11658	0.41071	0.0375
0.00641	0.01174	0	0.07832	0.21429	0.05
0	0	0	0	0	0
0.02564	0.0137	0	0.19126	0.60714	0.025

Table S3

	ACC	BLCA	BRCA	CESC	CHOL	COAD	DLBC	ESCA
ADAMTS1	0	0.00971	0.01116	0.02076	0	0.05013	0.02703	0.02174
ADAMTS2	0.03261	0.02427	0.01014	0.02422	0.01961	0.12281	0.02703	0.04891
ADAMTS3	0.02174	0.03398	0.00913	0.03114	0	0.06015	0.02703	0.03261
ADAMTS4	0.01087	0.01942	0.00609	0.0173	0	0.04261	0	0.02717
ADAMTS5	0	0.02427	0.01014	0.02768	0	0.08521	0.02703	0.02717
ADAMTS6	0.01087	0.01699	0.0071	0.02422	0	0.03008	0	0.02174
ADAMTS7	0.01087	0.01942	0.01318	0.02422	0	0.05764	0.02703	0.0163
ADAMTS8	0.01087	0.00971	0.00203	0.0173	0	0.0401	0	0.0163
ADAMTS9	0.01087	0.06068	0.01116	0.02422	0	0.07519	0.05405	0.03261
ADAMTS10	0.01087	0.0267	0.00203	0.02768	0	0.04511	0	0.00543
ADAMTS12	0.02174	0.06796	0.00811	0.04152	0.03922	0.08521	0	0.04348
ADAMTS13	0.02174	0.01699	0.01014	0.02768	0.01961	0.06015	0	0.02174
ADAMTS14	0.02174	0.0267	0.01116	0.02076	0	0.04762	0	0.0163
ADAMTS15	0	0.02184	0.00304	0.01384	0	0.03759	0	0.00543
ADAMTS16	0.03261	0.04126	0.01217	0.05536	0.03922	0.10025	0	0.05435
ADAMTS17	0.04348	0.01456	0.01217	0.0346	0	0.06266	0	0.0163
ADAMTS18	0.01087	0.04854	0.01116	0.04152	0	0.06767	0.05405	0.02717
ADAMTS19	0.03261	0.02184	0.01116	0.04152	0	0.03509	0	0.05435
ADAMTS20	0.01087	0.03883	0.0142	0.03806	0.01961	0.08772	0.02703	0.03804
ADAMTSL1	0	0.03883	0.02028	0.04844	0	0.07519	0.02703	0.04348
ADAMTSL2	0	0.02427	0.00406	0.02768	0	0.05263	0.05405	0
ADAMTSL3	0.01087	0.04854	0.0213	0.03806	0	0.09273	0	0.02717
ADAMTSL4	0.02174	0.01699	0.00507	0.03806	0	0.07268	0	0.0163
ADAMTSL5	0	0.00243	0.00203	0.00692	0	0.02256	0.02703	0.00543

GBM	HNSC	KICH	KIRC	KIRP	LAML	LGG	LIHC	LUAD
0.00769	0.00988	0.01515	0	0.00712	0.00746	0.00395	0.01099	0.03387
0.02051	0.01186	0.01515	0.01786	0.00712	0	0.00791	0.03022	0.07665
0.01538	0.03755	0	0.0119	0.00356	0	0.00791	0.02473	0.04635
0.00256	0.01976	0.01515	0.00595	0	0.00746	0.00198	0	0.02852
0.01026	0.02174	0	0.00893	0.00356	0	0.00198	0.01099	0.06952
0.00769	0.01383	0.01515	0.00298	0.01068	0	0.00593	0.00824	0.03209
0.01026	0.01779	0.01515	0.00893	0.01423	0	0.00198	0.01923	0.03565
0.00513	0.01581	0.01515	0.00595	0.00712	0	0.00198	0.00549	0.01426
0.01282	0.0336	0	0.00893	0.00356	0.00746	0.00988	0.02747	0.05169
0.01538	0.00988	0	0	0.01068	0	0.00198	0.01099	0.01783
0.0359	0.05336	0	0.02976	0.01068	0.01493	0.00988	0.03022	0.19251
0.00513	0.01186	0	0.00893	0.00712	0	0.00198	0.01648	0.02496
0.01026	0.02174	0	0.00893	0.01068	0	0.00395	0.01099	0.05169
0.00769	0.00791	0	0	0.00356	0	0.01383	0.00275	0.03209
0.03846	0.02964	0	0.00595	0.00712	0.00746	0.00791	0.01099	0.08378
0.01026	0.01186	0	0.00298	0.01068	0.00746	0.00593	0.00824	0.041
0.01795	0.02569	0	0.01488	0	0.00746	0.00198	0.01374	0.06595
0.01795	0.03557	0	0.0119	0.00356	0	0.00791	0.04121	0.05882
0.02308	0.0336	0	0.01488	0.01068	0	0.00395	0.03571	0.11765
0.01795	0.02767	0	0.00893	0	0	0.00791	0.02747	0.0713
0.00769	0.00395	0	0.00298	0	0	0.00198	0.00824	0.03209
0.01538	0.02372	0	0.00298	0.00712	0	0.00593	0.01648	0.082
0.00513	0.00791	0	0.00298	0.00356	0	0.00395	0.01923	0.02139
0.00769	0.00593	0	0.00298	0	0	0.00198	0.00824	0.00713

LUSC	MESO	OV	PAAD	PCPG	PRAD	READ	SARC	SKCM
0.01018	0	0.01147	0.00633	0	0.00207	0.03676	0.00422	0.00857
0.04277	0	0.00688	0.01266	0	0	0.02206	0.01266	0.0985
0.06721	0	0.00459	0.01266	0.00562	0.0062	0.08088	0.01688	0.05139
0.0387	0.0125	0.01606	0.01899	0	0.00207	0.02206	0	0.03854
0.04073	0	0.01147	0.01266	0	0.00826	0.02941	0	0.05567
0.0387	0	0.00917	0.00633	0	0.0062	0.03676	0.00844	0.17559
0.0387	0	0.00688	0.01266	0	0	0.02941	0.00422	0.10493
0.02444	0	0.00688	0.01266	0.00562	0.0062	0.01471	0.01266	0.05996
0.05092	0.0125	0.02294	0.00633	0	0.00826	0.02941	0.00422	0.11991
0.02648	0	0.00688	0.01266	0.00562	0.01033	0.03676	0	0.12848
0.1833	0	0.02294	0.03797	0	0.01033	0.07353	0.01266	0.12206
0.02851	0.0125	0.00688	0.00633	0	0.0062	0.02206	0	0.0621
0.02037	0.0125	0.00459	0.00633	0	0.00413	0.06618	0.00844	0.07066
0.02444	0	0	0.01899	0	0.0062	0.01471	0.00422	0.05353
0.10591	0	0.01606	0.02532	0.00562	0.01033	0.08824	0	0.11135
0.02648	0	0.01147	0.00633	0	0.00207	0.01471	0.01688	0.04069
0.0835	0.0125	0.00917	0.01899	0	0.00207	0.08088	0.02532	0.22698
0.05906	0	0.02752	0.02532	0	0.01033	0.02941	0.00844	0.13704
0.10591	0.0125	0.01835	0.00633	0.00562	0.01033	0.08824	0.03797	0.19914
0.04888	0	0.01835	0.01899	0.00562	0.01033	0.03676	0.01266	0.09636
0.01222	0	0.00459	0.01266	0	0.00413	0.00735	0.01266	0.05139
0.07332	0	0.01606	0.01899	0	0.00413	0.04412	0.01688	0.1242
0.01222	0.0125	0.00688	0.02532	0	0.00207	0.02206	0	0.05782
0.00611	0	0	0.00633	0	0.00207	0	0.00422	0.01285

STAD	TGCT	THCA	THYM	UCEC	UCS	UVM
0.04388	0	0	0	0.08507	0.01754	0
0.06236	0	0.00616	0	0.09074	0.03509	0
0.05774	0	0	0.0082	0.11531	0.03509	0
0.03695	0	0	0.01639	0.07561	0	0.0125
0.08545	0.0069	0	0	0.1172	0	0
0.03002	0	0.00205	0	0.09641	0	0
0.04619	0	0	0	0.0775	0.03509	0
0.03464	0	0.00411	0	0.0586	0.01754	0
0.06236	0	0	0.01639	0.11342	0	0
0.05543	0.0069	0	0	0.09452	0	0.0125
0.08776	0	0.00411	0	0.10586	0.01754	0.0125
0.0485	0	0	0.0082	0.10964	0.01754	0
0.05081	0	0	0	0.08507	0.01754	0
0.03926	0	0	0	0.05482	0	0
0.07852	0	0.00411	0.0082	0.12098	0.07018	0
0.05543	0	0	0	0.09074	0	0
0.09469	0	0.00411	0	0.09074	0.01754	0.0125
0.04388	0.0069	0.00205	0	0.09641	0.01754	0
0.04388	0.0069	0	0	0.11342	0.01754	0
0.05774	0.01379	0.00205	0	0.14367	0.08772	0.0125
0.03464	0	0.00205	0	0.07372	0.01754	0
0.06697	0	0.00205	0.0082	0.10775	0.01754	0
0.04619	0.01379	0.00205	0.01639	0.1172	0.01754	0
0.01155	0	0	0	0.02836	0	0

Table S4

	BLCA	COAD	ESCA	KICH	KIRC	KIRP	LIHC	LUAD
ADAMTS7	1.47902	0.96283	1.27807	0.60063	2.47557	1.54227	1.5531	0.3595
ADAMTS1	-3.14421	-1.79355	-1.7186	-2.2736	-0.30283	0.065	-0.9254	-2.17048
ADAMTS15	-1.26243	0.08073	-1.26321	-3.07792	-1.9965	-0.17151	0.71579	-1.39814
ADAMTS19	-0.71595	-0.1555	0.50617	-2.2107	-4.18328	-4.07559	3.38296	2.8026
ADAMTSL1	-1.30875	-1.96278	-1.78893	3.04453	-0.85265	-3.66195	0.64848	-0.71498
ADAMTS16	-1.3313	-0.32864	-0.49001	-1.44358	-2.61535	-0.49666	4.45792	2.49554
ADAMTS9	-1.57556	0.85787	-0.64223	-1.49634	0.2389	1.07917	1.50893	-0.55587
ADAMTSL4	-0.64535	-1.21076	-0.9098	0.74685	2.14632	1.43141	0.56802	-1.68173
ADAMTS12	1.90678	3.55752	3.79959	1.12222	1.34443	-0.40788	1.4007	1.98923
ADAMTSL3	-3.91425	-2.84276	-2.05516	-0.74664	0.16569	-0.56895	-0.28799	-2.17991
ADAMTS10	-0.49884	-0.09958	0.04517	-1.44163	2.2038	1.02701	1.64798	0.22618
ADAMTS8	-2.78518	-1.74065	-3.28763	-1.92785	-1.22525	-1.22177	0.55254	-3.43883
ADAMTS18	0.54677	1.5543	0.42242	1.29068	1.50266	-0.52184	3.80531	2.02979
ADAMTS20	3.1755	5.12981	2.56725	0.95235	5.41882	3.16906	4.72923	4.75571
ADAMTS17	-0.19776	-0.05891	-0.19854	0.7382	-0.34869	-1.41611	1.17437	-0.44567
ADAMTSL2	-0.29145	1.61559	1.10772	-1.77754	-1.28747	-2.41182	-0.86476	-0.48018
ADAMTS6	-0.27227	2.41285	0.24437	-2.0348	-0.53915	-2.4697	2.04507	0.91139
ADAMTSL5	-0.04933	1.3616	0.40837	0.0922	-0.05114	2.01511	1.40589	1.58623
ADAMTS4	-2.60008	1.19488	0.06059	-0.11308	1.98615	-1.37525	-0.19668	-1.11188
ADAMTS14	1.23365	1.23724	2.14981	1.3124	3.35115	3.50567	2.19356	2.76901
ADAMTS3	-0.22774	0.29973	0.40423	0.48563	-0.19611	1.31146	1.40274	0.60648
ADAMTS5	-1.62784	-0.22322	-0.34498	-0.2698	0.85185	-0.73017	0.82098	0.63882
ADAMTS2	0.81573	2.38886	1.57715	0.84303	1.91305	1.02585	0.10395	0.87979
ADAMTS13	-0.05427	0.62582	-0.6216	-0.35056	0.11978	1.0289	-2.18903	0.65362

LUSC	PCPG	PRAD	SKCM	STAD	THCA	UCEC
-0.24738	0.00652	0.2008	1.28174	1.33672	1.29462	1.3998
-1.72207	-0.59886	-0.53665	3.09593	-1.75167	-0.37084	-2.32845
-0.85715	0.05607	-0.70807	2.34813	-1.02714	0.03938	1.35706
1.61763	4.71856	0.03384	3.31046	2.00144	-0.84922	-0.41844
-0.25524	-2.23299	0.8419	1.01902	-1.58979	-0.07347	-2.5807
0.09691	1.3641	0.5129	5.53128	0.67409	0.17731	-1.44711
-1.58268	1.19041	-0.5059	1.95467	0.78699	2.62614	-0.33048
-2.18786	-1.39119	-0.73161	-0.077	-0.87468	0.41154	-2.47692
1.63125	0.54965	-0.15177	2.05286	3.4599	1.05582	-0.35384
-2.43845	-1.1494	-0.83451	-0.18242	-1.39125	-0.11346	-2.21345
-0.74025	-0.80091	-0.25918	-0.15146	0.2991	0.81195	-1.06933
-4.82332	0.08131	-0.74097	1.521	-1.37618	0.03817	1.37793
3.45274	1.92523	-2.52967	4.72658	3.89729	2.50694	1.45494
6.76812	1.74892	1.69585	2.79655	3.62684	0.92349	1.14152
1.34528	-1.05687	0.0613	1.06779	0.51203	0.69772	0.55641
-1.54011	-0.14152	0.28821	-0.04614	1.46459	0.80112	1.54425
-0.00564	1.18878	0.30444	0.86277	1.17008	0.55317	1.61387
2.01564	-0.57032	-0.62739	1.1029	0.3418	1.11632	-3.10802
-1.76872	1.31807	-0.19537	1.35861	0.0578	-0.13074	-2.34512
1.87039	3.14291	0.44897	2.09544	2.81178	3.68621	2.51489
0.24057	-0.37182	0.64646	1.36344	0.8516	-0.80775	-2.03026
0.40185	1.85028	-1.73248	2.65969	0.10739	-0.84544	-3.05539
0.78179	0.99957	0.78733	2.30425	3.07605	1.0694	-0.26087
1.05905	1.79935	0.66519	0.79042	-0.31441	-0.43896	0.35171

Table S5

	BLCA	COAD	ESCA	KICH	KIRC	KIRP	LIHC
ADAMTS7	3.91E-05	2.37E-07	0.001882	0.045334	2.91E-36	2.27E-09	3.68E-16
ADAMTS1	3.40E-12	7.84E-12	0.247671	2.15E-09	0.531401	0.851117	9.01E-10
ADAMTS15	2.14E-06	0.000997	0.068406	1.54E-11	1.60E-24	0.024878	0.991667
ADAMTS19	2.04E-06	6.11E-10	0.946818	0.000452	3.04E-24	6.84E-17	0.280241
ADAMTSL1	2.20E-07	3.41E-21	0.001431	6.58E-06	4.67E-23	2.61E-19	0.724803
ADAMTS16	0.030182	0.001433	0.838963	3.99E-09	9.12E-31	3.01E-05	1.68E-05
ADAMTS9	2.63E-05	1.72E-06	0.62905	5.28E-09	0.04326	7.63E-09	1.85E-11
ADAMTSL4	3.73E-05	1.00E-13	0.070452	0.009256	1.58E-29	1.83E-08	0.169319
ADAMTS12	0.003488	7.15E-22	1.43E-06	0.311381	3.96E-10	4.48E-06	0.00091
ADAMTSL3	7.21E-11	1.56E-19	0.071493	9.84E-08	0.284882	1.91E-08	5.91E-05
ADAMTS10	0.00073	0.048129	0.677091	1.44E-09	2.63E-27	0.004933	1.02E-09
ADAMTS8	1.10E-10	8.89E-09	0.002406	4.82E-12	1.02E-31	2.67E-11	0.047544
ADAMTS18	0.136674	0.010491	0.036722	0.269254	8.30E-13	2.89E-08	3.19E-10
ADAMTS20	0.000451	0.050421	0.003605	0.402572	1.28E-26	0.160896	2.86E-05
ADAMTS17	0.011399	0.030093	0.34238	0.64722	0.000101	2.10E-13	0.453151
ADAMTSL2	0.631505	8.70E-11	0.003126	2.48E-10	3.65E-27	2.30E-18	9.81E-12
ADAMTS6	0.127088	2.22E-16	0.068406	2.25E-11	1.35E-07	3.88E-14	6.23E-08
ADAMTSL5	0.845794	3.35E-14	0.079141	0.930014	0.000234	2.81E-06	1.82E-05
ADAMTS4	0.000223	6.21E-10	0.211553	0.181581	6.43E-23	1.89E-06	0.310997
ADAMTS14	0.075909	5.79E-11	5.17E-05	0.088063	1.87E-30	3.02E-11	1.68E-10
ADAMTS3	0.00305	0.678015	0.433687	0.490979	0.001879	7.20E-06	0.939757
ADAMTS5	7.55E-08	0.017749	0.875576	0.005164	4.60E-10	9.74E-10	0.024572
ADAMTS2	0.469563	1.86E-14	0.00184	0.147941	2.35E-25	0.022881	0.000345
ADAMTS13	0.860604	0.036337	0.122958	0.014473	0.073197	5.35E-05	5.33E-28

LUAD	LUSC	PCPG	PRAD	SKCM	STAD	THCA	UCEC
0.130188	0.321984	0.314095	0.275205	0.654375	3.44E-09	3.48E-19	0.000359
3.11E-24	1.07E-18	0.441416	0.011706	0.10961	3.34E-06	0.019759	2.05E-11
3.27E-22	1.29E-10	0.403381	1.67E-06	0.390513	8.14E-06	0.142578	0.23347
4.86E-05	0.000205	0.010094	0.142995	0.67948	0.421205	0.042095	0.223857
2.38E-05	0.012315	0.009	0.014544	0.757894	2.15E-11	0.192763	2.95E-08
3.98E-18	0.002656	0.135293	0.006221	0.144152	0.559486	0.000214	0.013588
0.000272	7.05E-15	0.10986	1.08E-06	0.542422	7.25E-06	1.52E-24	0.599895
2.37E-26	2.16E-27	0.007123	1.23E-06	0.462997	0.000771	0.214364	5.08E-12
1.77E-17	2.56E-10	0.531936	0.255386	0.255297	1.44E-16	0.000263	0.000209
5.75E-29	1.18E-26	0.016327	3.50E-08	0.370582	1.93E-05	0.006247	7.92E-09
0.962314	9.27E-10	0.066404	0.011034	0.362789	0.291389	2.77E-11	5.34E-08
2.58E-32	1.34E-30	0.346771	1.04E-06	0.332651	0.000223	0.059535	0.331194
8.72E-12	1.24E-07	0.199489	1.71E-13	0.276701	3.44E-13	2.40E-16	0.610563
1.66E-05	1.73E-24	0.921072	0.232979	0.964554	7.18E-06	0.019212	0.629926
2.10E-06	5.05E-05	0.024857	0.115702	0.820023	0.834253	1.04E-09	0.832764
1.09E-06	3.58E-22	0.567039	0.176251	0.467482	9.03E-07	2.28E-07	0.956567
0.007836	0.283641	0.360426	0.582442	0.665005	2.92E-07	0.04997	0.580116
6.60E-10	3.76E-14	0.040734	1.52E-08	0.58706	0.052624	0.000226	7.44E-11
0.539642	0.072636	0.797487	0.772899	0.820023	0.109049	0.017319	0.296334
9.69E-26	1.30E-15	0.083557	0.048021	0.476524	1.34E-16	4.66E-19	7.60E-08
0.526045	4.95E-05	0.204139	0.006972	0.797277	0.002232	7.84E-17	2.51E-10
0.501676	0.625948	0.098645	3.48E-17	0.19393	0.092579	2.59E-13	2.82E-08
0.000123	0.010429	0.984252	9.78E-05	0.237359	1.70E-15	0.006677	0.130892
0.045181	0.001054	0.006581	4.46E-07	0.71913	0.021151	1.48E-09	0.219531

Table S6

	ACC	BLCA	CHOL	COAD	DLBC	ESCA	KICH
ADAMTS1	9.47E-05	0.459781	0.909126	0.031083	0.940207	0.692284	0.187241
ADAMTS13	0.001421	0.031313	0.152101	0.068749	0.010793	0.261348	0.937441
ADAMTS3	0.178439	0.004518	0.604423	0.008018	0.587334	0.181499	0.464775
ADAMTS12	0.000772	0.006416	0.067553	0.207586	0.951284	0.883248	0.018137
ADAMTS20	0.547832	0.273188	0.47043	0.052289	0.248394	0.559126	0.019887
ADAMTSL1	0.0028	0.000789	0.043213	0.456876	0.701116	0.466017	0.552955
ADAMTS14	0.004821	0.00281	0.135959	0.202784	0.58085	0.382783	0.056064
ADAMTS17	0.710197	0.406653	0.374502	0.782988	0.301424	0.870065	0.725107
ADAMTSL2	0.054771	0.015828	0.732416	0.161526	0.940837	0.134421	0.082654
ADAMTS4	0.007777	0.188459	0.871025	0.023585	0.22795	0.89754	0.679891
ADAMTSL5	0.366335	0.100685	0.053447	0.259284	0.407456	0.112075	0.023019
ADAMTS2	0.004139	0.180228	0.753593	0.441941	0.842795	0.37656	0.419487
ADAMTS16	0.371168	5.83E-05	0.751598	0.001953	0.524707	0.269197	0.619248
ADAMTS7	0.003692	0.235848	0.484151	0.675718	0.109975	0.329463	0.056414
ADAMTSL3	0.724105	0.902627	0.864927	0.353374	0.462373	0.29252	0.258247
ADAMTS19	0.016747	0.815311	0.565903	0.245284	0.429695	0.340763	0.44227
ADAMTSL4	0.78133	0.007226	0.672201	0.021335	0.649985	0.074881	0.056553
ADAMTS6	0.006249	0.074686	0.011434	0.017625	0.794262	0.447018	0.014484
ADAMTS5	0.684534	0.120565	0.608535	0.063121	0.166703	0.539345	0.671455
ADAMTS15	0.096318	0.149643	0.322774	0.35156	0.791637	0.652385	0.473706
ADAMTS18	0.104122	0.00754	0.73492	0.052841	0.324294	0.482705	0.321041
ADAMTS10	0.674995	0.642148	0.658566	0.011206	0.608165	0.203158	0.645076
ADAMTS9	0.006856	0.001179	0.748849	0.329746	0.448184	0.631909	0.522699
ADAMTS8	0.192872	0.369397	0.168104	0.549901	0.483305	0.723866	0.38588

KIRC	KIRP	LAML	LIHC	LUAD	LUSC	MESO	OV
0.461861	0.12641	0.167174	0.353139	0.988238	0.549149	0.000308	0.790558
0.000253	0.321941	0.381814	0.907021	0.952651	0.94204	0.072754	0.665
0.000729	0.883787	0.868676	0.028164	9.31E-05	0.315445	0.117138	0.586107
0.000847	3.90E-07	0.275508	0.75373	0.170828	0.452156	0.002527	0.234561
0.577645	0.000517	0.198835	0.754981	0.040285	0.102573	0.467337	0.365687
0.722536	0.000947	0.71255	0.816834	0.86639	0.956806	0.698657	0.03651
9.23E-11	0.891657	0.361701	0.545556	0.502521	0.836978	0.072127	0.962961
0.62939	0.718351	0.029046	0.299389	0.213396	0.056872	0.069292	0.384109
0.035418	0.865791	0.454487	0.677723	0.419394	0.365502	0.824644	0.007098
0.0086	0.039075	0.770182	0.584759	0.83735	0.017599	0.55124	0.643338
4.26E-06	0.079257	0.180514	0.109377	0.511227	0.560466	1.52E-05	0.476261
0.003247	0.042785	0.351651	0.935804	0.068109	0.392128	0.251173	0.128132
0.299398	0.615335	0.258222	0.340225	0.648648	0.008436	0.645778	0.178356
0.55797	0.958068	0.03713	0.01502	0.075281	0.358971	0.09848	0.187995
0.037998	0.817571	0.658213	0.606693	0.747977	0.789055	0.146192	0.526097
0.158968	0.819104	0.370737	0.159682	0.60291	0.223739	0.528925	0.85024
0.00017	0.022204	0.732933	0.45723	0.591973	0.121729	0.055835	0.13665
8.22E-09	6.27E-05	0.568306	0.213146	0.000408	0.482155	0.007097	0.408258
0.131768	0.415168	0.467238	3.69E-07	0.346644	0.010472	1.36E-06	0.496939
0.020215	0.498765	0.008685	0.942423	0.079691	0.769126	0.728596	0.53433
0.848387	0.979637	0.020643	0.155369	0.510588	0.955353	0.958393	0.189848
5.29E-07	0.155053	0.129621	0.171595	0.21297	0.98724	0.571319	0.038345
0.252922	0.351069	0.96308	0.280627	0.030945	0.185045	0.473979	0.915471
0.04796	0.307205	0.270288	0.205015	0.004858	0.159148	0.108461	0.70532

PAAD	PCPG	PRAD	READ	SARC	SKCM	STAD	TGCT
0.510987	0.393696	0.241758	0.689684	0.854982	0.714588	0.138358	0.823045
0.784655	0.847287	0.000532	0.016088	0.012085	0.874382	0.186973	0.298334
0.383446	0.156155	0.500584	0.411245	0.174053	0.403423	0.09982	0.959336
0.189783	0.387032	0.690875	0.354652	0.26639	0.781075	0.049319	0.74083
0.001385	0.770748	0.663383	0.69857	0.207317	0.538769	0.900951	0.668086
0.100921	0.331208	0.565176	0.136106	0.92284	0.57328	0.386152	0.884032
0.099646	0.393795	0.120548	0.853947	0.563472	0.369954	0.427385	0.763595
0.030018	0.088247	0.000375	0.898332	0.001802	0.96693	0.674019	0.643394
0.150417	0.025546	0.609724	0.070113	0.347646	0.684568	0.232651	0.428702
0.688662	0.951185	0.346224	0.853805	0.481352	0.007133	0.695771	0.45749
0.789882	0.142829	0.787631	0.316783	0.823197	0.046552	0.384354	0.130298
0.64395	0.032857	0.701576	0.284346	0.496411	0.078265	0.32042	0.938593
0.167782	0.005812	0.45062	0.550382	0.549247	0.842448	0.924101	0.706281
0.481715	0.698687	0.825586	0.485823	0.640711	0.073195	0.239028	0.595093
0.366513	0.769537	0.454373	0.526704	0.184385	0.088207	0.366759	0.979082
0.407569	0.415365	0.24213	0.697355	0.122849	0.910598	0.815164	0.437447
0.836683	0.66997	0.962376	0.166587	0.941138	0.890776	0.63177	0.977615
0.149653	0.246736	0.760504	0.548676	0.351153	0.571222	0.407397	0.585445
0.474045	0.812817	0.583727	0.785248	0.455634	0.309743	0.032626	0.847828
0.726478	0.291892	0.563153	0.079041	0.364262	0.007343	0.387405	0.574781
0.203548	0.764148	0.839812	0.235651	0.302712	0.633621	0.442057	0.764253
0.232384	0.171573	0.814394	0.963999	0.002155	0.198439	0.018349	0.898474
0.171005	0.931839	0.648398	0.760222	0.33198	0.020067	0.49492	0.848723
0.041822	0.455045	0.917729	0.895895	0.58408	0.624539	0.027349	0.600239

THCA	THYM	UCEC	UVM
0.003273	0.255862	0.668814	0.123941
4.57E-05	0.21514	0.532654	0.59371
0.247082	0.668245	0.480994	0.322431
0.075583	0.810529	0.557315	0.018374
0.524084	0.621798	0.620132	0.619956
0.352182	0.521634	0.682691	0.456877
0.029503	0.648989	0.830766	0.031389
0.144943	0.056596	0.262598	0.854329
0.43325	0.245477	0.863006	0.157363
0.000268	0.811206	0.065759	0.002085
0.837052	0.416896	0.993149	0.121032
0.009005	0.583812	0.28795	0.031181
2.45E-05	0.922775	0.188162	0.558882
0.359288	0.521731	0.991373	0.863823
0.373535	0.970634	0.085684	0.314191
0.568899	0.589814	0.733696	0.877708
0.004801	0.986328	0.486904	0.071776
0.001867	0.113256	0.732769	0.801969
0.027783	0.48407	0.520046	0.021955
0.757701	0.042881	0.051732	0.410526
0.960583	0.091215	0.239229	0.578877
0.616231	0.291573	0.963618	0.387473
0.065853	0.356978	0.484586	0.001133
0.395629	0.569828	0.752624	0.023158

Table S7

	ACC	BLCA	CHOL	COAD	DLBC	ESCA	KICH
ADAMTS1	9.47E-05	0.459781	0.909126	0.031083	0.940207	0.692284	0.187241
ADAMTS13	0.001421	0.031313	0.152101	0.068749	0.010793	0.261348	0.937441
ADAMTS3	0.178439	0.004518	0.604423	0.008018	0.587334	0.181499	0.464775
ADAMTS12	0.000772	0.006416	0.067553	0.207586	0.951284	0.883248	0.018137
ADAMTS20	0.547832	0.273188	0.47043	0.052289	0.248394	0.559126	0.019887
ADAMTSL1	0.0028	0.000789	0.043213	0.456876	0.701116	0.466017	0.552955
ADAMTS14	0.004821	0.00281	0.135959	0.202784	0.58085	0.382783	0.056064
ADAMTS17	0.710197	0.406653	0.374502	0.782988	0.301424	0.870065	0.725107
ADAMTSL2	0.054771	0.015828	0.732416	0.161526	0.940837	0.134421	0.082654
ADAMTS4	0.007777	0.188459	0.871025	0.023585	0.22795	0.89754	0.679891
ADAMTSL5	0.366335	0.100685	0.053447	0.259284	0.407456	0.112075	0.023019
ADAMTS2	0.004139	0.180228	0.753593	0.441941	0.842795	0.37656	0.419487
ADAMTS16	0.371168	5.83E-05	0.751598	0.001953	0.524707	0.269197	0.619248
ADAMTS7	0.003692	0.235848	0.484151	0.675718	0.109975	0.329463	0.056414
ADAMTSL3	0.724105	0.902627	0.864927	0.353374	0.462373	0.29252	0.258247
ADAMTS19	0.016747	0.815311	0.565903	0.245284	0.429695	0.340763	0.44227
ADAMTSL4	0.78133	0.007226	0.672201	0.021335	0.649985	0.074881	0.056553
ADAMTS6	0.006249	0.074686	0.011434	0.017625	0.794262	0.447018	0.014484
ADAMTS5	0.684534	0.120565	0.608535	0.063121	0.166703	0.539345	0.671455
ADAMTS15	0.096318	0.149643	0.322774	0.35156	0.791637	0.652385	0.473706
ADAMTS18	0.104122	0.00754	0.73492	0.052841	0.324294	0.482705	0.321041
ADAMTS10	0.674995	0.642148	0.658566	0.011206	0.608165	0.203158	0.645076
ADAMTS9	0.006856	0.001179	0.748849	0.329746	0.448184	0.631909	0.522699
ADAMTS8	0.192872	0.369397	0.168104	0.549901	0.483305	0.723866	0.38588

KIRC	KIRP	LAML	LIHC	LUAD	LUSC	MESO	OV
0.461861	0.12641	0.167174	0.353139	0.988238	0.549149	0.000308	0.790558
0.000253	0.321941	0.381814	0.907021	0.952651	0.94204	0.072754	0.665
0.000729	0.883787	0.868676	0.028164	9.31E-05	0.315445	0.117138	0.586107
0.000847	3.90E-07	0.275508	0.75373	0.170828	0.452156	0.002527	0.234561
0.577645	0.000517	0.198835	0.754981	0.040285	0.102573	0.467337	0.365687
0.722536	0.000947	0.71255	0.816834	0.86639	0.956806	0.698657	0.03651
9.23E-11	0.891657	0.361701	0.545556	0.502521	0.836978	0.072127	0.962961
0.62939	0.718351	0.029046	0.299389	0.213396	0.056872	0.069292	0.384109
0.035418	0.865791	0.454487	0.677723	0.419394	0.365502	0.824644	0.007098
0.0086	0.039075	0.770182	0.584759	0.83735	0.017599	0.55124	0.643338
4.26E-06	0.079257	0.180514	0.109377	0.511227	0.560466	1.52E-05	0.476261
0.003247	0.042785	0.351651	0.935804	0.068109	0.392128	0.251173	0.128132
0.299398	0.615335	0.258222	0.340225	0.648648	0.008436	0.645778	0.178356
0.55797	0.958068	0.03713	0.01502	0.075281	0.358971	0.09848	0.187995
0.037998	0.817571	0.658213	0.606693	0.747977	0.789055	0.146192	0.526097
0.158968	0.819104	0.370737	0.159682	0.60291	0.223739	0.528925	0.85024
0.00017	0.022204	0.732933	0.45723	0.591973	0.121729	0.055835	0.13665
8.22E-09	6.27E-05	0.568306	0.213146	0.000408	0.482155	0.007097	0.408258
0.131768	0.415168	0.467238	3.69E-07	0.346644	0.010472	1.36E-06	0.496939
0.020215	0.498765	0.008685	0.942423	0.079691	0.769126	0.728596	0.53433
0.848387	0.979637	0.020643	0.155369	0.510588	0.955353	0.958393	0.189848
5.29E-07	0.155053	0.129621	0.171595	0.21297	0.98724	0.571319	0.038345
0.252922	0.351069	0.96308	0.280627	0.030945	0.185045	0.473979	0.915471
0.04796	0.307205	0.270288	0.205015	0.004858	0.159148	0.108461	0.70532

PAAD	PCPG	PRAD	READ	SARC	SKCM	STAD	TGCT
0.510987	0.393696	0.241758	0.689684	0.854982	0.714588	0.138358	0.823045
0.784655	0.847287	0.000532	0.016088	0.012085	0.874382	0.186973	0.298334
0.383446	0.156155	0.500584	0.411245	0.174053	0.403423	0.09982	0.959336
0.189783	0.387032	0.690875	0.354652	0.26639	0.781075	0.049319	0.74083
0.001385	0.770748	0.663383	0.69857	0.207317	0.538769	0.900951	0.668086
0.100921	0.331208	0.565176	0.136106	0.92284	0.57328	0.386152	0.884032
0.099646	0.393795	0.120548	0.853947	0.563472	0.369954	0.427385	0.763595
0.030018	0.088247	0.000375	0.898332	0.001802	0.96693	0.674019	0.643394
0.150417	0.025546	0.609724	0.070113	0.347646	0.684568	0.232651	0.428702
0.688662	0.951185	0.346224	0.853805	0.481352	0.007133	0.695771	0.45749
0.789882	0.142829	0.787631	0.316783	0.823197	0.046552	0.384354	0.130298
0.64395	0.032857	0.701576	0.284346	0.496411	0.078265	0.32042	0.938593
0.167782	0.005812	0.45062	0.550382	0.549247	0.842448	0.924101	0.706281
0.481715	0.698687	0.825586	0.485823	0.640711	0.073195	0.239028	0.595093
0.366513	0.769537	0.454373	0.526704	0.184385	0.088207	0.366759	0.979082
0.407569	0.415365	0.24213	0.697355	0.122849	0.910598	0.815164	0.437447
0.836683	0.66997	0.962376	0.166587	0.941138	0.890776	0.63177	0.977615
0.149653	0.246736	0.760504	0.548676	0.351153	0.571222	0.407397	0.585445
0.474045	0.812817	0.583727	0.785248	0.455634	0.309743	0.032626	0.847828
0.726478	0.291892	0.563153	0.079041	0.364262	0.007343	0.387405	0.574781
0.203548	0.764148	0.839812	0.235651	0.302712	0.633621	0.442057	0.764253
0.232384	0.171573	0.814394	0.963999	0.002155	0.198439	0.018349	0.898474
0.171005	0.931839	0.648398	0.760222	0.33198	0.020067	0.49492	0.848723
0.041822	0.455045	0.917729	0.895895	0.58408	0.624539	0.027349	0.600239

THCA	THYM	UCEC	UVM
0.003273	0.255862	0.668814	0.123941
4.57E-05	0.21514	0.532654	0.59371
0.247082	0.668245	0.480994	0.322431
0.075583	0.810529	0.557315	0.018374
0.524084	0.621798	0.620132	0.619956
0.352182	0.521634	0.682691	0.456877
0.029503	0.648989	0.830766	0.031389
0.144943	0.056596	0.262598	0.854329
0.43325	0.245477	0.863006	0.157363
0.000268	0.811206	0.065759	0.002085
0.837052	0.416896	0.993149	0.121032
0.009005	0.583812	0.28795	0.031181
2.45E-05	0.922775	0.188162	0.558882
0.359288	0.521731	0.991373	0.863823
0.373535	0.970634	0.085684	0.314191
0.568899	0.589814	0.733696	0.877708
0.004801	0.986328	0.486904	0.071776
0.001867	0.113256	0.732769	0.801969
0.027783	0.48407	0.520046	0.021955
0.757701	0.042881	0.051732	0.410526
0.960583	0.091215	0.239229	0.578877
0.616231	0.291573	0.963618	0.387473
0.065853	0.356978	0.484586	0.001133
0.395629	0.569828	0.752624	0.023158

Table S8

gene	conMean	treatMean	logFC	pValue
ADAMTSL3	2.94572	3.3042246	0.16569	0.284882487
ADAMTS12	0.34763	0.8827278	1.34443	3.96E-10
ADAMTS4	2.25145	8.919761	1.98615	6.43E-23
ADAMTS2	1.12304	4.2294267	1.91305	2.35E-25
ADAMTSL5	0.33053	0.3190188	-0.05114	0.000233679
ADAMTS18	0.13646	0.3866734	1.50266	8.30E-13
ADAMTS7	0.34072	1.8950454	2.47557	2.91E-36
ADAMTSL1	1.37149	0.759484	-0.85265	4.67E-23
ADAMTS19	0.28378	0.0156202	-4.18328	3.04E-24
ADAMTS20	0.00105	0.0448934	5.41882	1.28E-26
ADAMTS3	0.75459	0.6586802	-0.19611	0.001878915
ADAMTS8	0.76671	0.3279417	-1.22525	1.02E-31
ADAMTS16	3.3486	0.5464671	-2.61535	9.12E-31
ADAMTS10	0.7203	3.3183777	2.2038	2.63E-27
ADAMTS1	28.5856	23.173111	-0.30283	0.53140068
ADAMTS9	3.93979	4.649325	0.2389	0.043259921
ADAMTS15	6.29095	1.5765582	-1.9965	1.60E-24
ADAMTSL4	0.74816	3.3120918	2.14632	1.58E-29
ADAMTS14	0.06617	0.675237	3.35115	1.87E-30
ADAMTS17	0.35111	0.2757218	-0.34869	0.000100869
ADAMTS5	2.50869	4.5277074	0.85185	4.60E-10
ADAMTS13	0.51494	0.5595206	0.11978	0.07319711
ADAMTSL2	9.8377	4.0301933	-1.28747	3.65E-27
ADAMTS6	0.25179	0.1732775	-0.53915	1.35E-07

Table S9

id	HR	HR.95L	HR.95H	pvalue
age	1.0297	1.01588	1.04372	2.19E-05
grade	2.28263	1.84053	2.83093	5.73E-14
stage	1.92576	1.67699	2.21144	1.60E-20
T	1.97255	1.66095	2.34262	9.65E-15
M	4.49933	3.25415	6.22096	9.22E-20
riskScore	1.38284	1.27059	1.50501	6.18E-14

Table S10

id	HR	HR.95L	HR.95H	pvalue
age	1.0374	1.02184	1.0532	1.92E-06
grade	1.3644	1.06486	1.74821	0.0140159
stage	1.75006	1.14943	2.66456	0.009074
T	0.82171	0.55951	1.20678	0.3166182
M	1.39213	0.72455	2.6748	0.3207473
riskScore	1.31139	1.18438	1.45201	1.83E-07
