# Supplementary figures and images for: A New Survival Model Based on ADAMTSs for Prognostic Prediction in Clear Cell Renal Cell Carcinoma
Source: J Oncol. 2021 Sep 23;2021:2606213. doi: 10.1155/2021/2606213 (PMC8486512; doi:10.1155/2021/2606213)

risk 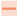  $>21.72(11)$  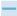  $<21.72(13)$

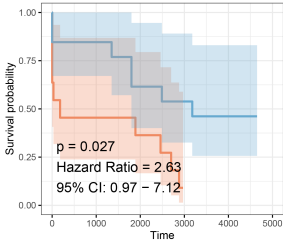

Supplement: Supplementary Materials — Figure S1: alternations of gene expression of ADAMTSs across cancer types. A: alternations of gene expression of ADAMTSs across cancer types. B-C: changes of gene expression of ADAMTS20 and ADAMTS14 in different types of cancer. E-F: the protein expression level of ADAMTS20 in KIRC and normal renal tissue. Figure S2: classical pathway analysis and drug sensitivity analysis of ADAMTSs. A-B: classical pathway analysis of ADAMTSs. C: drug sensitivity analysis of ADAMTSs. Figure S3: verification of the prognostic model based on ADAMTS (GSE22541). Figure S4: gene set enrichment analysis in KIRC. A: ADAMTSL2; B: ADAMTSL4; C: ADAMTS10; D: ADAMTS14. Table S1: CNV amplification frequency of ADAMTSs across cancer types. Table S2: CNV deletion frequency of ADAMTSs across cancer types. Table S3: SNV frequency of ADAMTSs across cancer types. Table S4: Log FCs of the expression of ADAMTSs across cancer types. Table S5: P values of the expression of ADAMTSs across cancer types. Table S6: HRs of ADAMTSs across cancer types. Table S7: P values of the HRs of ADAMTSs across cancer types. Table S8: the differential expression of ADAMTSs in KIRC and normal renal tissues. Table S9: univariate Cox regression analysis. Table S10: multivariate Cox regression analysis. . [file 2606213.f1.zip › 2606213.f1/FIG S3 (1).pdf]

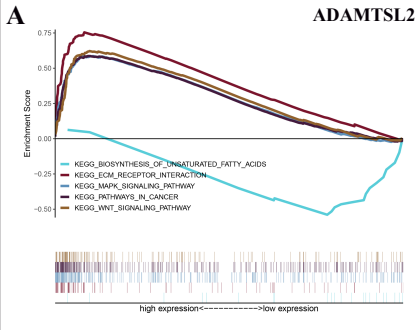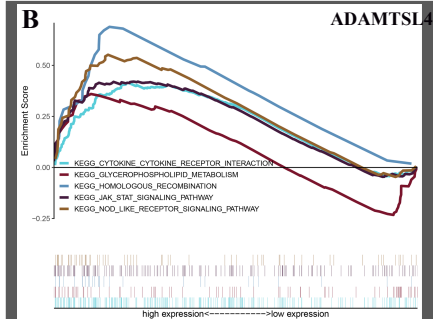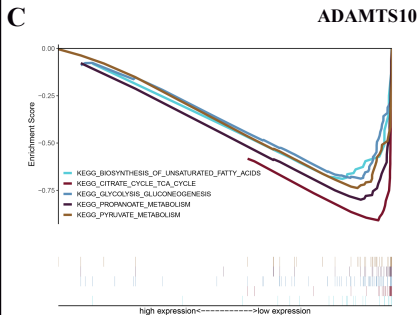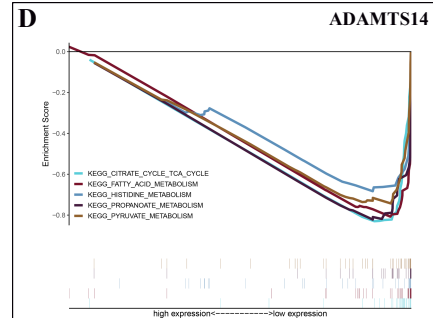

Supplement: Supplementary Materials — Figure S1: alternations of gene expression of ADAMTSs across cancer types. A: alternations of gene expression of ADAMTSs across cancer types. B-C: changes of gene expression of ADAMTS20 and ADAMTS14 in different types of cancer. E-F: the protein expression level of ADAMTS20 in KIRC and normal renal tissue. Figure S2: classical pathway analysis and drug sensitivity analysis of ADAMTSs. A-B: classical pathway analysis of ADAMTSs. C: drug sensitivity analysis of ADAMTSs. Figure S3: verification of the prognostic model based on ADAMTS (GSE22541). Figure S4: gene set enrichment analysis in KIRC. A: ADAMTSL2; B: ADAMTSL4; C: ADAMTS10; D: ADAMTS14. Table S1: CNV amplification frequency of ADAMTSs across cancer types. Table S2: CNV deletion frequency of ADAMTSs across cancer types. Table S3: SNV frequency of ADAMTSs across cancer types. Table S4: Log FCs of the expression of ADAMTSs across cancer types. Table S5: P values of the expression of ADAMTSs across cancer types. Table S6: HRs of ADAMTSs across cancer types. Table S7: P values of the HRs of ADAMTSs across cancer types. Table S8: the differential expression of ADAMTSs in KIRC and normal renal tissues. Table S9: univariate Cox regression analysis. Table S10: multivariate Cox regression analysis. . [file 2606213.f1.zip › 2606213.f1/FIG S4 (1).pdf]
