# Supplementary material for: A New Survival Model Based on ADAMTSs for Prognostic Prediction in Clear Cell Renal Cell Carcinoma
Source: J Oncol. 2021 Sep 23;2021:2606213. doi: 10.1155/2021/2606213 (PMC8486512; doi:10.1155/2021/2606213)
Supplement: Supplementary Materials — Figure S1: alternations of gene expression of ADAMTSs across cancer types. A: alternations of gene expression of ADAMTSs across cancer types. B-C: changes of gene expression of ADAMTS20 and ADAMTS14 in different types of cancer. E-F: the protein expression level of ADAMTS20 in KIRC and normal renal tissue. Figure S2: classical pathway analysis and drug sensitivity analysis of ADAMTSs. A-B: classical pathway analysis of ADAMTSs. C: drug sensitivity analysis of ADAMTSs. Figure S3: verification of the prognostic model based on ADAMTS (GSE22541). Figure S4: gene set enrichment analysis in KIRC. A: ADAMTSL2; B: ADAMTSL4; C: ADAMTS10; D: ADAMTS14. Table S1: CNV amplification frequency of ADAMTSs across cancer types. Table S2: CNV deletion frequency of ADAMTSs across cancer types. Table S3: SNV frequency of ADAMTSs across cancer types. Table S4: Log FCs of the expression of ADAMTSs across cancer types. Table S5: P values of the expression of ADAMTSs across cancer types. Table S6: HRs of ADAMTSs across cancer types. Table S7: P values of the HRs of ADAMTSs across cancer types. Table S8: the differential expression of ADAMTSs in KIRC and normal renal tissues. Table S9: univariate Cox regression analysis. Table S10: multivariate Cox regression analysis. . [file FIG S1 (1).pdf › 2606213.f1/FIG S1 (1).pdf]

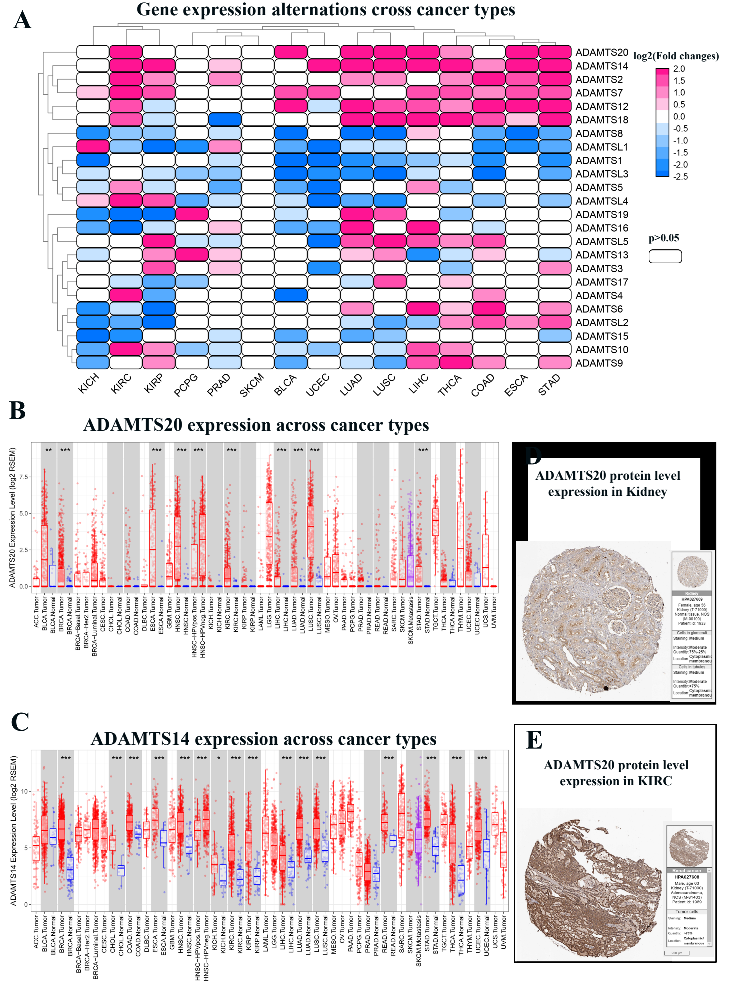
A
Gene expression alternations cross cancer types
ADAMTS20
ADAMTS14
ADAMTS2
ADAMTS7
ADAMTS12
ADAMTS18
ADAMTS8
ADAMTSL1
ADAMTS1
ADAMTSL3
ADAMTS5
ADAMTSL4
ADAMTS19
ADAMTS16
ADAMTSL5
ADAMTS13
ADAMTS3
ADAMTS17
ADAMTS4
ADAMTS6
ADAMTSL2
ADAMTS15
ADAMTS10
ADAMTS9
KICH
KIRC
KIRP
PCPG
PRAD
SKCM
BLCA
UCEC
LUAD
LUSC
LIHC
THCA
COAD
ESCA
STAD
log2(Fold changes)
2.0
1.5
1.0
0.5
0.0
-0.5
-1.0
-1.5
-2.0
-2.5
p>0.05
B
ADAMTS20 expression across cancer types
ADAMTS20 Expression Level (log2 RSEM)
D
ADAMTS20 protein level expression in Kidney
C
ADAMTS14 expression across cancer types
ADAMTS14 Expression Level (log2 RSEM)
E
ADAMTS20 protein level expression in KIRC
